# Prevalence of bovine tuberculosis in dairy cattle in China during 2010–2019: A systematic review and meta-analysis

**DOI:** 10.1371/journal.pntd.0009502

**Published:** 2021-06-17

**Authors:** Qing-Long Gong, Yu Chen, Tian Tian, Xiaobo Wen, Dong Li, Yu-Hao Song, Qi Wang, Rui Du, Xiao-Xuan Zhang

**Affiliations:** 1 College of Chinese Medicine Materials, Jilin Agricultural University, Changchun, Jilin Province, PR China; 2 College of Animal Science and Technology, Jilin Agricultural University, Changchun, Jilin Province, PR China; 3 College of Animal Science and Veterinary Medicine, Heilongjiang Bayi Agricultural University, Daqing, Heilongjiang Province, PR China; 4 College of Animal Science and Technology, Hainan University, Hainan Key Lab of Tropical Animal Reproduction and Breeding and Epidemic Disease Research, Haidian Island, Haikou, Hainan Province, PR China; 5 College of Veterinary Medicine, Qingdao Agricultural University, Qingdao, Shandong Province, PR China; Lowell General Hospital, UNITED STATES

## Abstract

**Background:**

Bovine tuberculosis (bTB), caused by members of the *Mycobacterium tuberculosis* complex bacteria, mainly *Mycobacterium bovis* (*M*. *bovis*), is a major threat to public health and economic development. There has been no systematic epidemiological assessment concerning bTB in dairy cattle in China.

**Methodology/principal findings:**

Literature related to bTB in China was retrieved from China National Knowledge Infrastructure (CNKI), PubMed, ScienceDirect, VIP Chinese Journals Database, and Wan Fang Database to build the first meta-analysis for estimating the prevalence and infection moderators of bTB in dairy cattle in China. A total of 100 relevant studies published from 2010 to 2019 were included. We estimated the overall prevalence of bTB was 2.4% (95% CI: 2.1–2.8) during this decade. In the sampling year subgroup, the prevalence was lowest in 2017 or later at 0.8% (95% CI: 0.3–1.5). The lowest prevalence was 0.7% (95% CI: 0.5–1.0) in Northwestern China. The lowest prevalence was 2.1% (95% CI: 1.8–2.5) using SIT test. Heifer cows had the highest prevalence, which was 27.1% (95% CI: 9.7–49.2). The prevalence in scale farming was 3.7% (95% CI: 3.1–4.3), significantly higher than that in free-range farming (1.7%, 95% CI: 1.1–2.4). The prevalence of bTB was highest in summer at 4.0% (95% CI: 1.7–7.0). In addition, the influence of different geographical factors (altitude, longitude, latitude, precipitation, temperature, humidity) on the prevalence was analyzed.

**Conclusions/significance:**

The results showed that bTB was widespread in China but has been gradually reduced through concerted national intervention. It is suggested that different countries should formulate corresponding prevention and control measures according to the epidemic situation in its cattle industry. Enhanced monitoring of warm and humid areas may play an important role in reducing the incidence of bTB. In addition, when large-scale breeding is promoted, attention should be paid to standardizing breeding management and improving animal welfare to reduce the prevalence of bTB in cattle.

## Author summary

bTB is a neglected zoonotic disease caused by members of the *Mycobacterium tuberculosis* complex bacteria (*M*. *bovis*, mainly), which is also called “TB disease from bovine sources” when it occurs in humans. It is still widespread in China although the prevalence has gradually reduced through national intervention. Because beef and dairy products are important human dietary sources of protein, bTB has a great impact on public health and safety. We constructed the first meta-analysis to assess the epidemic of bTB in dairy cattle in China over the past 10 years and analyzed the potential moderators of bTB. A total of 100 studies were included. The results showed that bTB was common and unevenly distributed in China. The potential moderators affecting the bTB epidemic were region, sampling year, detection methods, age of dairy cattle, feeding mode, season, and certain geographical and climatic factors (longitude, precipitation, mean temperature, humidity, and altitude). These important findings may help in the formulation of policies to control the prevalence of bTB in cattle, thereby reducing the economic losses caused by bTB and reducing the threat of bTB to human health.

## Introduction

Bovine tuberculosis (bTB), a chronic granulomatous inflammatory disease is caused mainly by *Mycobacterium bovis* (*M*. *bovis*) [1]. Via the respiratory or digestive tract, *M*. *bovis* can infect a wide range of hosts, including many common mammals such as cattle, humans, non-human primates, giraffes, seals, goats, cats, dogs, pigs, buffalo, badgers, possums, deer, and bison [2–4], but it also poses a major threat to some endangered species [5]. Before pasteurization of milk, *M*. *bovis* was deemed the leading cause of death in children from abdominal tuberculosis [6]. The disease poses a huge threat to public health and causes a related socioeconomic burden [7]. The World Health Organization (WHO) has estimated that about 143,000 (71,200–240,000) incident cases of zoonotic tuberculosis (caused by *M*. *bovis*) occurred globally in 2018 (https://apps.who.int/iris/handle/10665/329368) [8].

According to the FAO (2013), more than 6 billion people worldwide consume milk and dairy products. Milk from dairy cattle represent about 85% of the total milk produced worldwide [9]. Tuberculosis in cows results in low milk productivity, weight loss, infertility, mortality, and condemnation of carcasses. It is estimated that the global intended scope of loss to livestock industry due to bTB is about US$3 billion a year [10]. Some countries have attained bTB-free status through test-and-slaughter programs, while others have failed to eradicate the disease because of multiple epidemiological conditions such as the presence of other maintenance hosts for *M*. *bovis* [11,12]. However, the true burden of the disease is unknown in most developing countries, where lack of epidemiological data and high economic costs often hinder strategies to control transmission of *M*. *bovis* [13].

China ranks second among the 22 countries with a high burden of tuberculosis listed by WHO, behind only India [8]. By 2018, China had 14.8 million dairy cows, an increase of 4.1% over 2017 [14]. To date, China has issued a series of control measures to prevent and control bTB, such as establishing monitoring and early warning, emergency response, and other systems; supporting intensive breeding, guiding farmers to unify epidemic prevention; and implementing a disease eradication plan for breeding farms. However, data on the epidemiology of bTB in dairy cows in China are still incomplete. Therefore, we constructed the first meta-analysis to assess the prevalence of bTB and further analyzed the associated moderators, including geographical region and various other geographic factors, sampling year, detection methods, age, sampling season, and study quality level in an attempt to contribute to future prevention and control of the disease.

## Methods

### Search strategy

We performed this meta-analysis based on the PRISMA guidelines (Table A in S1 Text) [15,16]. The literature on the prevalence of bTB in dairy cattle in China published during 2010–2019 was retrieved from databases including PubMed, ScienceDirect, China National Knowledge Infrastructure (CNKI), VIP Chinese Journals Database, and Wan Fang Database. In PubMed, we used the MeSH terms “Cattle”, “Tuberculosis”, and “China” to retrieve medical subject headings and entry terms concerning them. Then Boolean operators “AND” and “OR” were used to connect medical subject headings and entry terms in an advanced search to generate the final search formula:

(“Cattle”[Mesh] OR Bos indicus OR Zebu OR Zebus OR Bos taurus OR Cow, Domestic OR Cows, Domestic OR Domestic Cow OR Domestic Cows OR Bos grunniens OR Yak OR Yaks)

AND (“Tuberculosis”[Mesh] OR Tuberculoses OR Kochs Disease OR Koch’s Disease OR Koch Disease OR *Mycobacterium tuberculosis* Infection OR Infection, *Mycobacterium tuberculosis* OR Infections, *Mycobacterium tuberculosis* OR *Mycobacterium tuberculosis* Infections)

AND (“China”[Mesh] OR People’s Republic of China OR Mainland China OR Manchuria OR Sinkiang OR Inner Mongolia).

In ScienceDirect, the keywords of “Cattle”, “Tuberculosis”, “prevalence”, and “China” and article type of “Research articles” were used to search. The same Chinese search formula with fuzzy search and synonym expansion was used in advanced search of the three Chinese databases (CNKI, Wan Fang, and VIP database):

(“tuberculosis” AND “cattle” OR “*Mycobacterium tuberculosis*” AND “cattle”)

To avoid missing valid literature, we also supplemented the advanced search results by using the terms “cow disease”, “cow tuberculosis”, “bovine tuberculosis” in CNKI and the term “cow tuberculosis” in the VIP with the default options.

### Selection criteria

Three reviewers (TT, DL, and YC) extracted data from qualified studies. In case of disagreement, the author (QLG) conducted further evaluation. We strictly selected qualified literature according to the following criteria: (1) It was a cross-sectional prevalence study. (2) Data concerned cattle in China. (3) The test was performed using standard bTB testing methods, including single intradermal test (SIT), enzyme-linked immunosorbent assay (ELISA), IFN-γ-ELISA, SIT&IFN-γ-ELISA, and colloidal gold test. (4) The study provided sample totals and prevalence. (5) The subjects were dairy cattle. (6) The full text was available.

### Data extraction

We used standardized forms in Microsoft Excel 2007 to collect the following information: first author, publication year, sampling year, geographical region, sampling season, age, detection method, feeding mode, total number of samples, and positive numbers.

### Quality assessment

According to the criteria derived from the Grading of Recommendations Assessment, Development and Evaluation method (GRADE) [17], we assessed the quality of the eligible publications. One point per criteria was awarded if the study met the following criteria: “clear detection objectives”, “clear detection methods”, “clear sampling time” and “four or more moderators”. A paper with a final score of 0–1 was low quality, 2 was medium quality, and 3–4 was high quality. The low-quality studies were eventually excluded from the analysis.

### Statistical analysis

We did all analyses using the “meta” package (version 4.12–0) in R software version 3.5.2 [18]. As suggested by previous studies, the Freeman-Tukey double arcsine transformation (named PFT the meta package) has better variance stabilization performance, therefore, we used PFT for rate conversion before meta to make the rate more consistent with the Gaussian distribution [19,20]. The formulas for PFT were as follows:

$$\begin{array}{l} t=arcsin\;(sqrt\;(r/(n+1)))+arcsin\;(sqrt\;(\left(r+1)/(n+1\right)))\\ se(t)=sqrt(1/(n+0.5))\\ p={\left(sin(t/2)\right)}^{2} \end{array}$$

Note: t: transformed prevalence; n = sample size; r = positive number; se = standard error.

To facilitate reporting, we reconverted the transformed summary proportion and its confidence interval [18]. The variation was quantified using the I^2^, Cochran’s Q, and χ^2^ tests. A random-effects model was used to perform the meta-analysis due to the expected strong heterogeneity. The publication bias was evaluated by the funnel plot, trim and fill analysis, and Egger’s test. It has been shown that different subgroups may generate different funnel plots because prevalence changes over time [21]. Therefore, each subgroup was used to generate funnel plots and forest plots for further assessment. The stability of the study was determined by sensitivity analysis. Forest plots are used to present results visually. The code in R for this meta-analysis is shown in Table B in S1 Text.

Subgroup analysis and univariate meta-regression analysis were used to reveal factors that may contribute to heterogeneity among studies. The factors included geographical region (comparison of Northern China with others), sampling year (comparison of 2012 or before with other groups), detection methods (comparison of SIT with other groups), age (comparison of heifer with others), sampling season (comparison of summer with others), quality level (comparison of high with others). In addition, in order to reduce the heterogeneity caused by different detection methods, when a study used multiple detection methods, we pooled the data that came from the SIT method (because most of the studies we included used SIT).

China has a vast territory and significant climatic differences between different regions. Thus, we also assessed the impact of different geographical factors on the study, including latitude (comparison of 30–35° with other groups), longitude (comparison of >110° with other groups), average annual precipitation (comparison of >1500 mm with other groups), average annual temperature (comparison of 10–15°C with other groups), average annual humidity (comparison of >70% with other groups), altitude (comparison of <1000 m with other groups). All surface meteorological data are from China Integrated Meteorological Information Sharing System (CIMISS). For sampling sites without weather stations, we selected weather station data from nearby counties through high-resolution maps. In case that was unavailable as well, meteorological station data from the nearest city were selected. For sampling sites without sampling years, we extracted data concerning only the altitude, longitude, and latitude. In addition, correlation analysis was conducted for each subgroup with detection methods and provinces respectively to trace the source of heterogeneity. The explained heterogeneity of the covariates is expressed in R^2^.

## Results

### Search results and eligible studies

**Fig 1:**
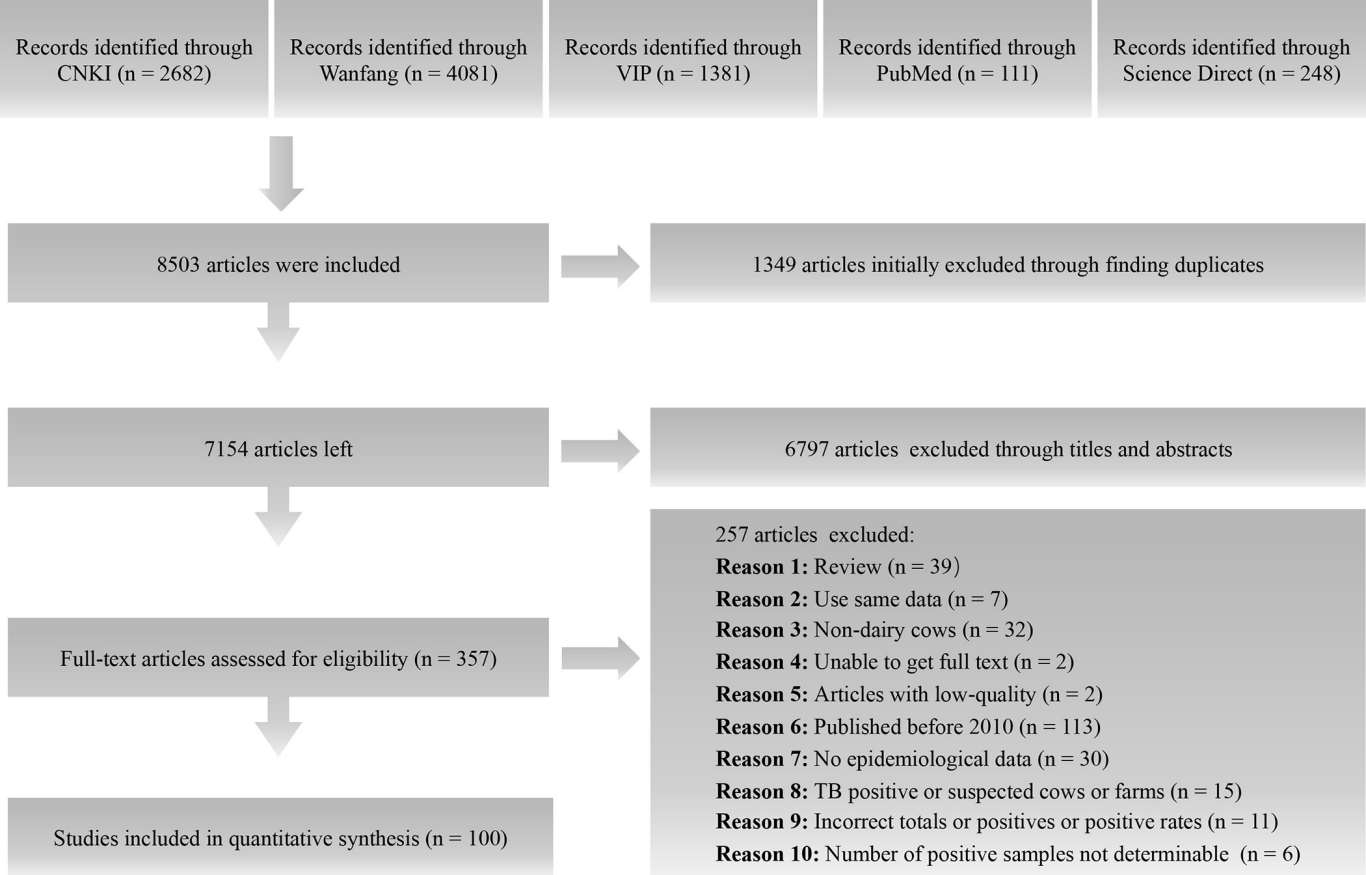
Flow diagram of eligible studies for searching and selecting.

**Fig 2:**
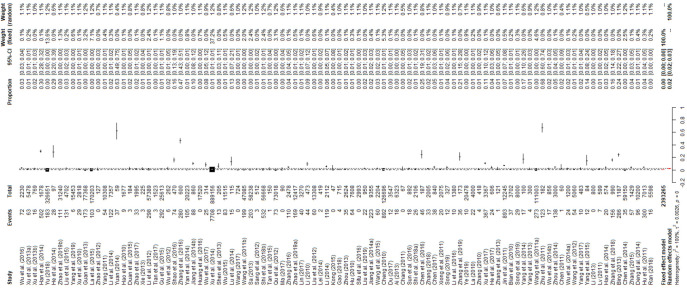
Forest plot of bovine tuberculosis prevalence among studies conducted in China. The horizontal line represents the 95% confidence interval, and the diamond represents the summarized effect.

According to the inclusion and exclusion criteria, there were two low-quality papers (which were excluded), 15 medium-quality papers, and 85 high-quality papers; ultimately, 100 included studies were used for meta-analysis (Figs 1 and 2 and Tables 1 and 2, and Table C in S1 Text).

**Table 1:** Included studies of bTB infection in dairy cattle in China.

Study ID	Sampling time	Province	Detection method ^a^	bacterial isolation	Positive samples/total samples	Prevalence	Quality score
**Central China**							
Chen (2011)	UN ^b^	Hunan	SIT	Yes	1/60	1.67%	2
Li et al. (2014)	2010–2013	Hubei	SIT	No	122/7,357	1.66%	4
Chen (2017)	2015.03–2016.10	Henan	SIT	No	38/840	4.52%	4
Zhao et al. (2019a)	2018	Henan	SIT	No	169/12,417	1.36%	3
**Eastern China**							
Li (2010)	UN	Shandong	SIT	No	44/473	9.30%	3
Zhang (2010)	2009.08	Jiangxi	SIT	No	4/418	0.96%	4
Hu et al. (2011)	2007–2010	Zhejiang	SIT	No	300/7,013	4.28%	3
Jin et al. (2011)	2003–2010	Zhejiang	SIT	No	893/12,245	7.29%	4
Wang et al. (2011a)	2003–2009	Fujian	SIT	No	273/111,003	0.25%	3
Qu et al. (2012)	2007–2011(exclude2009)	Shanghai	SIT	No	73/73,018	0.10%	4
Shen et al. (2013)	UN	Shanghai	SIT	No	13/205	2.08%	3
Xu et al. (2013b)	2009–2011	Jiangsu	SIT	No	16/769	29.92%	4
Han et al. (2014)	2010–2012	Shandong	SIT	No	502/1,678	3.48%	4
Hao et al. (2014)	2012.06–2013.06	Shandong	SIT	No	20/574	2.38%	4
Jiang et al. (2014a)	2010–2012	Shanghai	SIT	No	223/9,355	0.87%	4
Xu et al. (2014)	2007–2013	Jiangsu	SIT	No	7,708/889,156	9.30%	4
Duan et al. (2015)	UN	Zhejiang	SIT	No	3/184	1.63%	3
Gu et al. (2015)	2011–2013	Jiangsu	SIT	No	292/25,613	1.14%	4
Yang et al. (2015)	UN	Shanghai	SIT&IFN-γ-ELISA	No	12/84	14.29%	3
Chen et al. (2016)	2014.08–2015.02	Shandong	Test strip	No	46/187	24.60%	4
Sun et al. (2016)	2013–2015	Jiangsu	SIT	No	18/892	2.02%	4
Yang et al. (2017)	UN	Shanghai	SIT	No	10/483	2.07%	3
Tian et al. (2017)	2016.03	Shandong	SIT	No	3/1,523	0.20%	4
Wu et al. (2017)	2015.07	Shanghai	SIT	No	25/314	7.96%	4
**Northern China**							
Zhao et al. (2012)	UN	Beijing	SIT	No	0/127	0.00%	3
Yang et al. (2013)	UN	Beijing	SIT	No	4/300	1.33%	2
Li (2014)	UN	Inner Mongolia	ELISA	No	119/2,112	5.63%	3
Lu et al. (2014)	UN	Hebei	SIT	No	15/115	13.04%	3
Wang et al. (2015)	UN	Hebei	SIT	No	60/2,204	2.72%	2
Huang et al. (2016)	2015	Beijing	SIT	No	0/17,520	0.00%	4
Zhang (2016)	UN	Inner Mongolia	ELISA	No	110/2,478	4.44%	2
Song et al. (2019)	2017.10–2018.06	Beijing	SIT	No	29/8,000	0.36%	4
Zhang et al. (2019)	2017.06	Beijing	IFN-γ-ELISA	No	36/173	20.81%	4
**Northeastern China**							
Zhang (2018)	2014–2017	Liaoning	SIT	No	33/2,005	1.65%	3
Liu et al. (2019)	UN	Heilongjiang	SIT	No	22/950	2.32%	3
Song (2019)	UN	Heilongjiang	ELISA	No	12/327	3.67%	2
**Northwestern China**							
Cheng (2010)	2003–2008	Xinjiang	SIT	No	892/126,696	0.70%	3
Hao et al. (2010)	2007.04–2007.06	Qinghai	SIT	No	9/1,677	0.54%	4
Hu (2010)	2006–2009	Qinghai	SIT	No	64/7,608	0.84%	3
La (2010)	2009.06–2009.09	Qinghai	SIT	No	22/4,000	0.55%	3
Chang (2011)	UN	Gansu	SIT	No	0/67	0.00%	3
Lv (2011)	2009.04–2009.06	Qinghai	SIT	No	7/599	1.17%	3
Wang et al. (2011b)	2007–2010	Xinjiang	SIT	No	199/47,085	0.42%	3
Yang (2011)	2008.06–2010.07	Qinghai	SIT	No	54/10,308	0.52%	4
Li et al. (2012)	2007–2011	Xinjiang	SIT	No	298/57,389	0.52%	4
Liu et al. (2012)	2008–2011	Qinghai	SIT	No	60/13,308	0.45%	4
Sang et al. (2012)	2010	Xinjiang	SIT	No	3/512	0.59%	4
Wu et al. (2012)	2011.06–2011.07	Xinjiang	SIT	No	6/262	2.29%	3
Ai (2013)	2011.01–2013.05	Xinjiang	SIT	No	18/6,323	0.28%	4
Guan et al. (2013)	2010–2012	Xinjiang	SIT	No	173/77,368	0.22%	3
Li (2013)	2013.7	Qinghai	SIT	No	0/800	0.00%	3
Sa (2013)	UN	Xinjiang	SIT	No	203/58,238	0.35%	3
Xie (2013)	2012.04–2012.06	Qinghai	SIT	No	3/225	1.33%	4
Zhang et al. (2013)	UN	Shaanxi	SIT	No	1/121	0.83%	2
Zhou (2013)	UN	Xinjiang	SIT	No	35/2,624	1.33%	2
Chen et al. (2014)	2008–2012	Qinghai	SIT	No	35/59150	0.06%	3
Lei (2014)	2012.04–2012.06	Qinghai	SIT	No	2/419	0.48%	4
Zhou et al. (2014)	UN	Xinjiang	SIT	No	138/3,000	4.60%	2
Kong (2015)	UN	Gansu	SIT	No	0/47	0.00%	2
La et al. (2015)	2009–2014	Qinghai	SIT	No	103/170,203	0.06%	3
Liu et al. (2015)	2012–2015	Xinjiang	SIT	No	131/4,702	2.79%	4
Tan et al. (2015)	UN	Ningxia	SIT	No	1/150	0.67%	3
Wu (2015)	2010–2014	Shaanxi	SIT	No	83/11,515	0.72%	4
Zhang (2015)	2015.02–2015.05	Ningxia	SIT	No	57/1,429	3.99%	3
Li et al. (2016)	2015.09–2015.10	Qinghai	SIT	No	10/1,380	0.72%	4
Wu et al. (2016)	2015	Ningxia	SIT	No	72/2,230	3.23%	4
He et al. (2017)	2014–2016	Shaanxi	SIT	No	24/606	3.96%	3
Lin (2017)	2015.06–2015.11	Qinghai	SIT	No	40/3,270	1.22%	4
Ma (2017)	2016.02	Qinghai	SIT	No	0/90	0.00%	4
Wang (2017)	2016.03	Qinghai	SIT	No	0/724	0.00%	4
Zhao et al. (2017)	2015	Shaanxi	SIT	No	33/1,995	1.65%	3
Gao (2018)	2016.06–2017.10	Shaanxi	SIT	No	4/715	0.56%	4
Shi et al. (2018a)	UN	Xinjiang	SIT	No	26/2,106	1.23%	2
Shi et al. (2018b)	2012–2018.06	Xinjiang	SIT	No	532/56,668	0.94%	3
Yu (2018)	2011–2016	Gansu	SIT	No	183/326,651	0.06%	3
**Southern China**							
Xu et al. (2010)	2010	Guangxi	SIT	No	29/2,818	1.03%	3
Xu et al. (2013a)	2008–2011	Guangxi	SIT	No	63/5,478	1.15%	4
Jiang et al. (2014b)	2003–2013	Guangxi	SIT	No	88/860	10.23%	3
Yan et al. (2014)	2004.01–2013.12	Guangxi	SIT	No	105/20,223	0.52%	4
Situ et al. (2016)	2012–2015	Guangdong	SIT	No	0/2,993	0.00%	4
**Southwestern China**							
Bian et al. (2010)	2007–2009	Sichuan	SIT	No	37/3,702	1.00%	3
Xiong et al. (2011)	2010.02–2010.03	Yunnan	SIT	No	20/2,075	0.96%	4
Wang et al. (2012)	2010–2011	Yunnan	SIT	No	54/5,060	1.07%	4
Deng et al. (2014)	UN	Sichuan	SIT	No	96/10,200	0.94%	2
He et al. (2014)	2009.06–2009.08	Yunnan	SIT	No	28/97	28.87%	4
Wu et al. (2014a)	UN	Guizhou	SIT	No	24/1,200	2.00%	3
Yang et al. (2014)	UN	Guizhou	SIT	No	17/100	17.00%	3
Du (2017)	2016.06–2016.11	Yunnan	SIT	No	12/3,547	0.34%	4
Ran (2018)	2013–2017	Sichuan	SIT	No	16/5,598	0.29%	4
Yang et al. (2019)	2016–2017	Sichuan	SIT&IFN-γ-ELISA	No	6/15,453	0.04%	3
Ye (2019)	2017–2018	Guizhou	IFN-γ-ELISA	No	10/20,478	0.05%	4
Zhao et al. (2019b)	2012–2018	Yunnan	SIT	No	111/31,240	0.36%	3
**Missing**							
Zhu et al. (2011)	UN	UN	SIT	No	123/182	67.58%	2
Bing (2013)	2011.10–2012.06	UN	IFN-γ-ELISA	Yes	286/1,187	24.09%	4
Wu et al. (2014b)	2012	UN	SIT	No	17/856	1.99%	4
Yuan (2014)	UN	UN	SIT	No	37/59	62.71%	3
Shao et al. (2016)	UN	UN	IFN-γ-ELISA	No	74/470	15.74%	2
Zhang et al. (2016)	UN	UN	SIT	No	280/600	46.67%	3
Xu et al. (2017)	UN	UN	SIT	No	367/3,367	10.90%	2
Zhang et al. (2018)	UN	UN	IFN-γ-ELISA	No	156/990	15.76%	2

^a^Detection methods: SIT: single intradermal test (SIT); ELISA: enzyme-linked immunosorbent assay, detection antibody in serum; IFN-γ-ELISA: detection of IFN-γ in whole blood; SIT&IFN-γ-ELISA: SIT is use for the first detection, and the positive samples obtained from SIT are tested again using IFN-γ-ELISA.

^b^UN: Unclear.

**Table 2:** Pooled prevalence and potential infection moderators of bTB infection in dairy cattle in China.

		No. studies	No. tested	No. positive	% (95% CI) ^a^	Heterogeneity			Univariate meta-regression		Correlation Analysis ^c^	
						*χ*^*2*^	*P*	*I*^*2*^ (%)	*P* ^b^	Coefficient (95% CI)	R^2^-methods	R^2^-provinces
Region ^d^	Northern China	19	33,029	373	3.2% (1.1–6.3)	994.75	0.00	99.2%	< 0.001	0.084 (0.059 to 0.108)	0.00%	26.13%
	Central China	4	20,674	330	2.0% (1.2–3.0)	31.47	< 0.01	90.5%				
	Eastern China	20	1,135,187	10,478	3.9% (2.9–5.0)	5986.55	0.00	99.7%				
	Northeastern China	3	3,282	67	2.3% (1.4–3.3)	5.68	0.06	64.8%				
	Northwestern China	39	1,062,260	3,528	0.7% (0.5–1.0)	4106.18	0.00	99.1%				
	Southern China	5	32,372	285	1.5% (0.4–3.1)	296.12	< 0.01	98.6%				
	Southwestern China	12	98,750	431	1.1% (0.6–1.6)	528.58	< 0.01	97.9%				
Sampling year	2012 or before	44	1,696,615	12,841	1.6% (1.2–1.9)	11141.71	0.00	99.6%	0.003	0.031 (0.011 to 0.052)	0.00%	40.80%
	2013–2016	30	521,530	1,618	0.9% (0.6–1.2)	2866.28	0.00	99.0%				
	2017 or later	8	62,760	300	0.8% (0.3–1.5)	438.27	< 0.01	98.4%				
Detection methods ^e^	ELISA	3	4,917	241	4.8% (3.8–5.8)	4.44	0.11	55.0%	< 0.001	-0.149 (-0.189 to -0.110)	2.49%	19.85%
	IFN-γ-ELISA	5	23,298	562	12.7% (0.9–34.8)	1899.76	0.00	99.8%				
	SIT	89	2,349,200	16,108	2.1% (1.8–2.5)	18294.77	0.00	99.5%				
	SIT&ELISA	2	15,537	18	-	-	-	-	-	-	-	-
	Colloidal gold test	1	187	46	-	-	-	-	-	-	-	-
Age ^f^	Calf	5	17,991	150	11.5% (0.0–45.9)	735.99	< 0.01	99.5%	< 0.001	0.256 (0.134 to 0.378)	0.00%	5.51%
	Heifer	4	747	175	27.1% (9.7–49.2)	111.03	< 0.01	97.3%				
	Adult cow	17	209,974	1,093	6.7% (4.1–9.8)	3625.75	0.00	99.6%				
	Young cow	1	47	15	-	-	-	-	-	-	-	-
Feeding mode	Free-range	15	179,062	677	1.7% (1.1–2.4)	826.34	< 0.01	98.3%	0.008	0.048 (0.012 to 0.083)	0.00%	30.14%
	Scale	62	1,633,651	13,649	3.7% (3.1–4.3)	15594.09	0.00	99.6%				
Season ^g^	Spring	8	16,123	105	0.8% (0.1–2.1)	278.90	< 0.01	97.5%	0.013	0.104 (0.022 to 0.187)	0.00%	41.78%
	Summer	10	15,241	185	4.0% (1.7–7.0)	349.45	< 0.01	97.4%				
	Autumn	5	26,673	915	1.4% (0.0–6.6)	1746.09	0.00	99.8%				
	Winter	4	29,180	116	0.4% (0.0–1.3)	83.83	< 0.01	96.4%				
Quality level	Medium	15	28,476	1203	5.9% (3.1–9.3)	1481.57	0.00	99.1%	< 0.001	-0.050 (-0.072 to -0.028)	1.94%	28.00%
	High	85	2,364,789	15,622	2.2% (1.9–2.5)	18024.12	0.00	99.5%				
Total		100	2,393,265	16,825	2.4% (2.1–2.8)	20446.92	0.00	99.5%				

^a^ Confidence interval.

^b^ P < 0.05 is statistically significant.

^c^ Correlation analysis: Joint analysis with prevalence of detection methods and provinces of China; R^2^: Proportion of between-study variance explained.

^d^ Northern China: Beijing, Hebei, Inner Mongolia; Northwestern China: Gansu, Ningxia, Qinghai, Shaanxi, Xinjiang; Southwestern China: Guizhou, Sichuan, Yunnan; Northeastern China: Heilongjiang, and Liaoning; Central China: Hubei, Henan, Hunan; Eastern China: Fujian, Jiangsu, Jiangxi, Shandong, Shanghai, Zhejiang; Southern China: Guangdong, Guangxi.

^e^ ELISA: SIT: single intradermal test (SIT); ELISA: enzyme-linked immunosorbent assay, detection antibody in serum sample; IFN-γ-ELISA: detection of IFN-γ in whole blood sample; SIT&IFN-γ-ELISA: SIT is use for the first detection, and the positive samples obtained from SIT are tested again using IFN-γ-ELISA.

^f^ Calf: 0–6 months old; Young cow: 7–15 months old; Heifer: 16 months old to first delivery; Adult cow: after first delivery.

^g^ Spring: March through May; Summer: June through August; Autumn: September through November; Winter: December through February.

### Publication bias and sensitivity analysis

**Fig 3:**
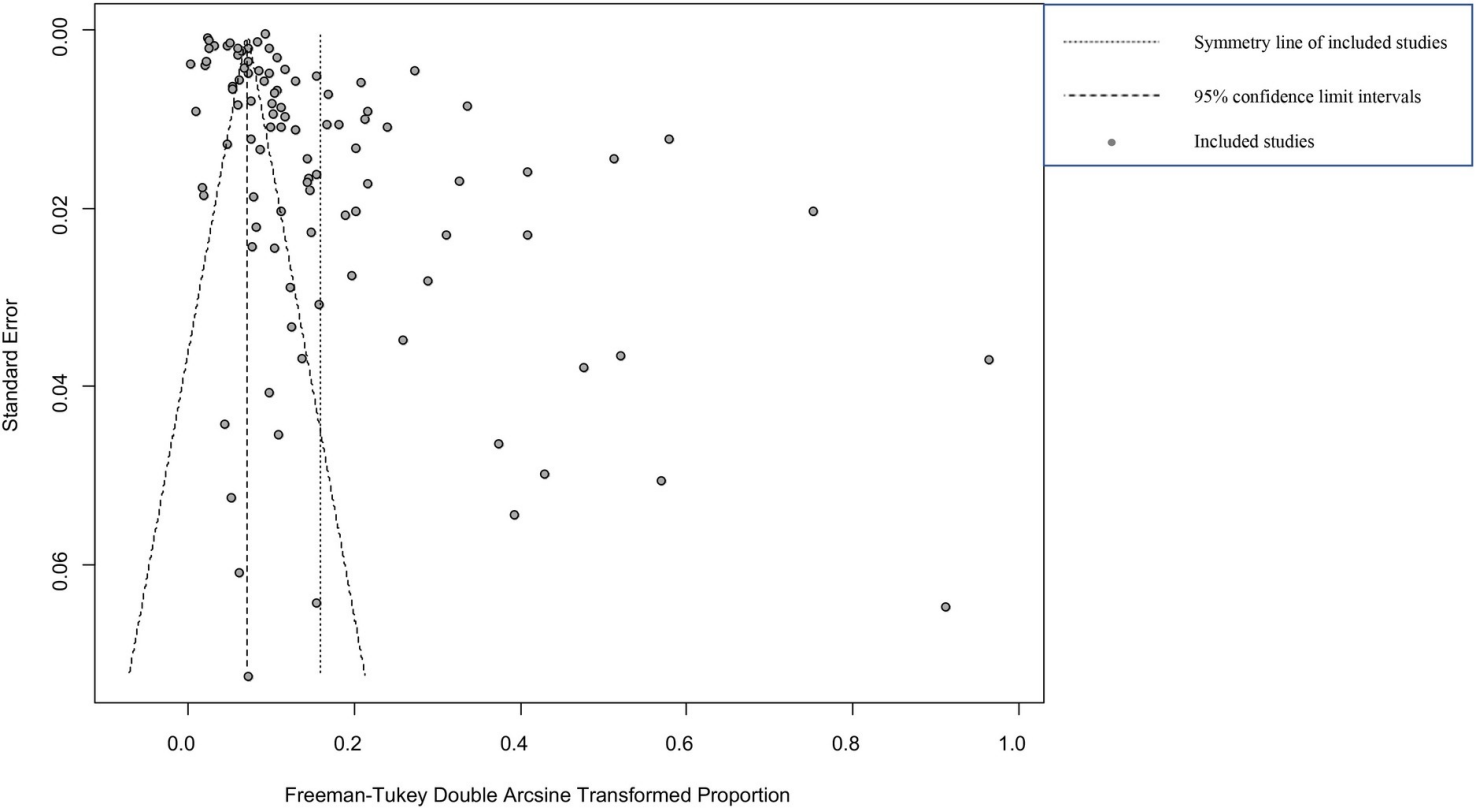
Funnel plot with pseudo 95% confidence limits intervals for the examination of publication bias.

**Fig 4:**
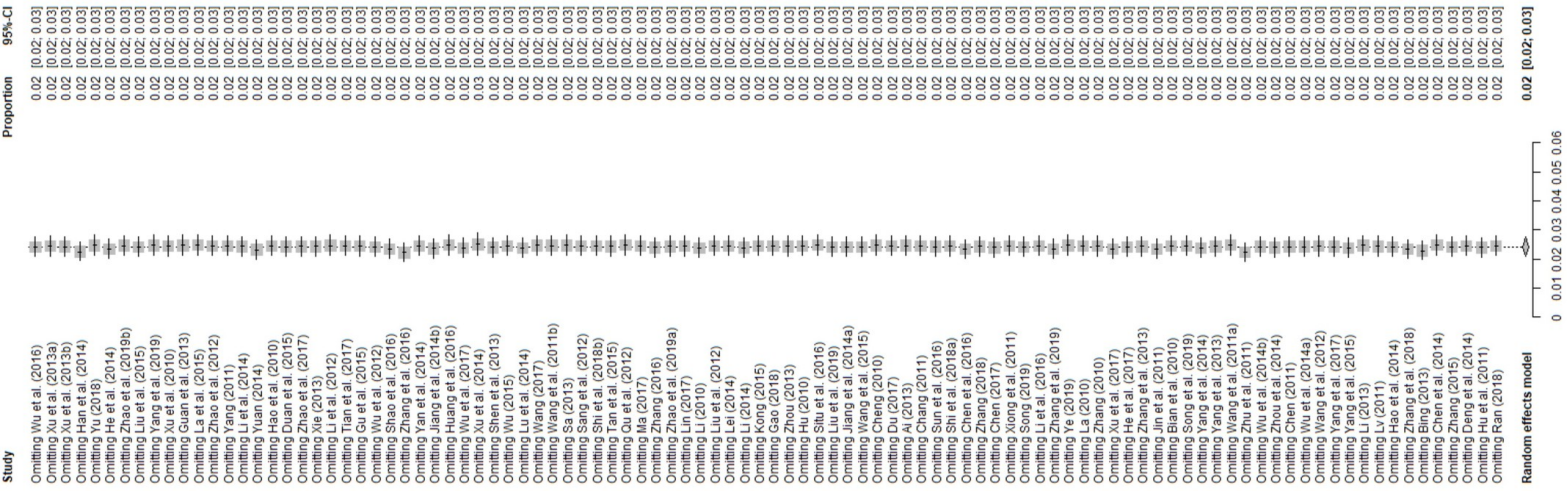
Sensitivity analysis. After removing one study at a time, the remaining studies were re-combined using a random-effects model to verify the impact of a single study on the overall results.

The forest plot shows great heterogeneity between selected studies (Fig 2). The funnel plot indicates that there may be publication bias or small-sample bias in our studies (Fig 3). Heterogeneity was further quantified using Egger’s test, which showed that publication bias did exist between studies (P = 0.0001; S1 Fig and Table D in S1 Text). We used trim and fill analysis to evaluate the impact of publication bias on the study results. The estimated value of the combined effect size did not change significantly after supplementing part of the study and performing the meta-analysis again, which indicated that publication bias had little impact and the results were relatively robust (S2 Fig). The meta-analysis results and publication bias of each subgroup are shown in the Supplementary Figures (S3–S16 Figs). In sensitivity analysis, each study removed had little impact on the result, indicating that our results were stable (Fig 4).

### Pooling analyses

As suggested by previous studies, we finally chose PFT to perform rate conversion (Table E in S1 Text) [19]. The overall prevalence of *M*. *bovis* among dairy cattle in China was 2.4% (95% CI 2.1–2.8, 16,825/2,393,265; Fig 2 and Table 2).

### Bovine tuberculosis infection in China

The highest prevalence was in Eastern China, and the lowest was in Northwestern China (Table 2). In terms of province level, the prevalence was highest in Shandong, while Gansu and Guangdong had the lowest prevalence (Table 3). Based on data from the *Chinese Animal Husbandry and Veterinary Yearbook* in 2018, we estimated the number of dairy cattle infected with *M*. *bovis* in China at 259,176 (226,779–302,372) (Table 4).

**Table 3:** Pooled prevalence of tuberculosis infection in dairy cattle in different provinces of China.

Province	Region	Study No.	No. tested	No. positive	% (95% CI)	Heterogeneity			Univariate meta-regression	
						χ^2^	P	I^2^ (%)	P	Coefficient (95% CI)
Beijing	Northern China	5	26,120	69	1.5% (0.3–3.6)	224.98	< 0.01	98.2%	< 0.001	0.199 (0.155 to 0.243)
Fujian	Eastern China	1	111,003	273	0.3% (0.2–0.3)	0.00	< 0.01	--		
Gansu	Northwestern China	3	326,765	183	0.0% (0.0–0.0)	0.82	< 0.01	0.0%		
Guangdong	Southern China	1	2,993	0	0.0% (0.0–0.1)	0.00	< 0.01	--		
Guangxi	Southern China	4	29,379	285	2.2% (0.7–4.4)	226.36	< 0.01	98.7%		
Guizhou	Southwestern China	3	21,778	51	3.2% (0.1–9.7)	130.06	< 0.01	98.5%		
Hebei	Northern China	2	2,319	75	6.7% (0.2–20.3)	18.95	< 0.01	94.7%		
Heilongjiang	Northeastern China	2	1,277	34	2.8% (1.6–4.2)	1.72	< 0.01	41.9%		
Henan	Central China	2	13,257	207	2.7% (0.4–6.7)	30.57	< 0.01	96.7%		
Hubei	Central China	1	7,357	122	1.7% (1.4–2.0)	0.00	< 0.01	--		
Hunan	Central China	1	60	1	1.7% (0.0–7.0)	0.00	< 0.01	--		
Inner Mongolia	Northern China	2	4,590	229	5.0% (3.9–6.2)	3.42	< 0.01	70.8%		
Jiangsu	Eastern China	4	916,430	8,034	1.2% (0.9–1.6)	36.93	< 0.01	91.9%		
Jiangxi	Eastern China	1	418	4	1.0% (0.2–2.2)	0.00	< 0.01	--		
Liaoning	Northeastern China	1	2,005	33	1.7% (1.1–2.3)	0.00	< 0.01	--		
Ningxia	Northwestern China	3	3,809	137	3.2% (2.1–4.5)	5.85	< 0.01	65.8%		
Qinghai	Northwestern China	15	273,761	409	0.4% (0.2–0.6)	463.97	< 0.01	97.0%		
Shaanxi	Northwestern China	5	14,952	145	1.3% (0.5–2.5)	42.79	< 0.01	90.7%		
Shandong	Eastern China	5	4,435	615	10.4% (0.6–29.3)	970.11	< 0.01	99.6%		
Shanghai	Eastern China	6	83,459	356	3.9% (1.3–7.6)	676.09	< 0.01	99.3%		
Sichuan	Southwestern China	4	34,953	155	0.5% (0.1–1.2)	177.59	< 0.01	98.3%		
Xinjiang	Northwestern China	13	442,973	2,654	0.9% (0.7–1.3)	917.03	< 0.01	98.7%		
Yunnan	Southwestern China	5	42,019	225	1.6% (0.7–2.8)	142.03	< 0.01	97.2%		
Zhejiang	Eastern China	3	19,442	1,196	4.5% (2.4–7.2)	84.62	< 0.01	97.6%		

^a^ Correlation analysis: Joint analysis with prevalence of provinces of China; R^2^: Proportion of between-study variance explained; I^2^-res: Residual variation due to heterogeneity.

**Table 4:** Estimated number of bTB infection in dairy cattle in China.

Region	Estimated number of dairy cattle in China*	Prevalence of tuberculosis infection	Estimated number of dairy cattle with tuberculosis infection
Northern China	3,012,000	3.2% (1.1–6.3)	96,384 (33,132–189,756)
Northeastern China	1,656,000	2.3% (1.4–3.3)	38,088 (23,184–54,648)
Eastern China	1,368,000	3.9% (2.9–5.0)	53,352 (39,672–68,400)
Central China	439,000	2.0% (1.2–3.0)	8,780 (5,268–13,170)
Southern China	111,000	1.5% (0.4–3.1)	1,665 (444–3,441)
Southwestern China	1,405,000	1.1% (0.6–1.6)	15,455 (8,430–22,480)
Northwestern China	2,808,000	0.7% (0.5–1.0)	19,656 (14,040–28,080)
Total	10,799,000	2.4% (2.1–2.8)	259,176 (226,779–302,372)

*Estimates of the number of dairy cattle in each region were obtained from 2018 data of the *Chinese Animal Husbandry and Veterinary Yearbook*.

### Other moderators associated with bTB

Our analysis manifested that region, sampling year, detection method, age, feeding mode, sampling season, and article quality level are related moderators (P < 0.05). Among them, the prevalence in 2012 or before was much higher than in the other two time periods. Compared with other detection methods, IFN-γ-ELISA yields higher values of the prevalence. The prevalence of bTB in heifers was highest. The prevalence of bTB in cattle with scale breeding was higher than that in free-range breeding. The prevalence was much higher in summer than in other seasons. Medium-quality articles showed the highest prevalence (Table 2). The heterogeneity of each subgroup explained by detection methods (the covariate) ranged from 0–2.49% (R^2^-methods), and that explained by provinces (the covariate) was 5.51%–41.78% (R^2^-provinces) (Table 2).

After subgroup analysis of geographical factors, we found that the prevalence in the groups of latitude 30–35°, longitude >110°, average annual precipitation >1500 mm, average annual temperature 10–15°C, average annual humidity 70%, altitude < 100 m was significantly higher than in the corresponding other groups (P < 0.05; Table 5).

**Table 5:** Geographical and climatic factors of bTB infection in dairy cattle in China.

		No. studies	No. tested	No. positive	% (95% CI)	Heterogeneity			Univariate meta-regression	
						*χ*^*2*^	*P*	*I*^*2*^ (%)	*P*-value^a^	Coefficient (95% CI)
Latitude	20–30°	18	205,658	1,956	2.12% (1.15–3.35)	3,203.38	0.00	99.5	0.032	0.029 (0.002 to 0.056)
	30–35°	9	113,111	645	2.13% (1.10–3.47)	869.66	< 0.001	99.1		
	35–40°	39	151,475	707	0.97% (0.63–1.37)	1,263.15	< 0.001	97.0		
	40–50°	15	359,074	2,270	0.70% (0.50–0.92)	506.56	< 0.001	97.2		
Longitude	85–100°	11	355,819	2,179	0.47% (0.32–0.64)	351.08	< 0.001	97.2	< 0.001	0.072 (0.049 to 0.095)
	100–105°	29	145,833	515	0.49% (0.27–0.74)	528.35	< 0.001	94.7		
	105–110°	11	50,418	466	1.46% (0.87–2.20)	281.83	< 0.001	96.5		
	> 110°	30	277,248	2,418	2.74% (1.88–3.74)	4,683.20	0.00	99.4		
Precipitation (mm)	< 400	18	108,437	954	0.95% (0.65–1.30)	293.13	< 0.001	94.2	0.029	0.060 (0.006 to 0.114)
	400–1000	24	144,549	562	1.40% (0.91–1.98)	1,079.93	< 0.001	97.9		
	1000–1500	12	143,723	804	1.26% (0.69–1.98)	955.16	< 0.001	98.8		
	> 1500	6	133,618	1,496	3.31% (0.44–8.40)	2,832.49	0.00	99.8		
Mean temperature (°C)	< 10	29	409,072	2,357	0.50% (0.34–0.68)	854.05	< 0.001	96.7	< 0.001	0.068 (0.034 to 0.101)
	10–15	10	38,703	324	3.46% (1.69–5.80)	853.18	0.00	98.9		
	15–20	18	284,168	2,268	2.03% (1.23–3.03)	3,763.88	< 0.001	99.5		
	> 20	6	29,554	256	1.85% (0.51–3.90)	309.94	< 0.001	98.4		
Humidity (%)	40–50	13	127,493	721	0.92% (0.61–1.29)	258.61	< 0.001	95.4	< 0.001	0.059 (0.031 to 0.087)
	50–60	23	315,819	1,857	0.59% (0.33–0.90)	1,285.71	< 0.001	98.3		
	60–70	9	64,106	388	1.55% (0.92–2.33)	280.51	< 0.001	97.1		
	> 70	18	254,079	2,239	2.67% (1.62–3.96)	4,001.01	0.00	99.6		
Altitude (m)	< 100	27	177,844	1,974	2.66% (1.65–3.88)	4,039.47	0.00	99.4	< 0.001	0.058 (0.034 to 0.081)
	100–1000	10	130,537	554	2.05% (1.07–3.32)	546.50	< 0.001	98.4		
	1000–2000	29	453,477	2,936	0.69% (0.54–0.86)	791.94	< 0.001	96.5		
	> 2000	15	67,460	114	0.35% (0.04–0.89)	192.77	< 0.001	92.7		

^a^Amount of heterogeneity accounted.

^b^Estimated amount of residual heterogeneity.

## Discussion

Bovine TB is a global zoonotic disease, which has caused huge economic losses and serious public health problems [22]. Therefore, a deeper understanding of bTB epidemiology is crucial for future prevention and control. Our assessment of the prevalence of bTB in dairy cattle in China showed that the prevalence was 2.4%, lower than in the developing country India (7.3%) and comparable to that in the developed country Spain (2.87%), which reflects the high degree of control of bTB in China in the past decade. In the year subgroup, the prevalence of 2013–2016 was significantly lower than that of 2012 or before (P < 0.05). In May 2012, China launched the National Plan for the Prevention and Control of Medium and Long-term Animal Epidemics (2012–2020), which classified bTB as a class II animal epidemic and formulated comprehensive prevention and control measures [23]. In June 2013, the International Cooperation Committee of Animal Welfare (ICCAW) of China was established to facilitate cooperation with regard to experience and information on farm animal welfare at home and abroad and to introduce international advanced welfare farming concepts and technology to China. National disease control and information exchange with foreign countries have played a positive role in reducing bTB [24]. The prevalence has been lowest since 2017. In June 2017, the Ministry of Agriculture issued the National Guidelines for Controlling Bovine TB among Dairy Cattle (2017–2020) [25], which adopted comprehensive prevention and control measures such as quarantine culling, risk assessment, movement control, and strengthening health management for domestic dairy cows. The policy aims to strengthen international cooperation with the FAO, the World Organisation for Animal Health (OIE), and other organizations (2017). In December of the same year, the OIE, the WHO, the FAO, and the International Union Against Tuberculosis and Lung Disease (The Union) jointly launched the first-ever roadmap to tackle zoonotic TB [26]. Continuous reduction in prevalence of bTB has been the result of a concerted national and global effort. Targeted prevention and control policies (such as the timely culling of sick cattle) and comprehensive policy implementation may be the key to prevention and control of bTB. In addition, livestock production systems in developed countries are primarily based on intensive dairy farming as opposed to extensive small-scale production systems in developing countries which are known to have low prevalence of bTB. Therefore, another main reason for the low prevalence of bTB in China may be due to the extensive production system.

In the subgroup of feeding mode, the prevalence of scale cultivation was significantly higher than that of free-range (P < 0.05). More intuitively, except for Xinjiang Province, the areas with high prevalence of dairy cow tuberculosis in China were generally concentrated in provinces with large dairy cow populations. Intensive farming has been shown to be a contributing factor to bTB in animals [14,27]. It is well known that bTB can be transmitted through the respiratory tract, which is exacerbated by the closure of cattle sheds on scale farms, and higher stocking density also tends to be associated with regional epidemic of bTB [28]. The relatively high levels of sunlight outside, lower farming density, and better air circulation in free-range cattle reduce the burden of *M*. *bovis*, relative to that in scale farming [29]. In recent years, mass movement of dairy cattle between large-scale farms in China and the introduction of foreign cattle breeds may also accelerate the spread of bTB [30]. However, we still need to treat this result with caution because the funnel plot indicates publication bias in this subgroup. In general, there are still deficiencies in intensive farming. Therefore, we suggest standardizing the breeding and management, improving the environment of the barn, and increasing animal welfare when developing the scale breeding of cows, which may have a better prevention and control effect [31].

Understanding age-specific risks is critical to accurately interpreting trends in pooled epidemiological data and to designing and evaluating control strategies such as vaccination [32]. Almost all previous studies have shown an increase in infection rates with age, and the relationship is generally monotonic or linear, with a U-shaped relationship reported [33]. However, our study showed that the prevalence was significantly different at different age stages and decreased with age (P < 0.001). It is worth noting that the sample size of the calf, young cow, and heifer groups is much smaller than that of the adult cow group, so small-study effects may lead to unstable results. Moreover, the funnel plot of this subgroup also suggests publication bias. Therefore, the relationship between the two requires further study.

In the seasonal subgroup, the prevalence in summer was significantly higher than in winter (P < 0.05). Cattle are more stressed in the hotter and wetter summer months [28,31]. In addition, the days are longer than the nights in summer, giving cows that are active for a prolonged time more opportunity to come into contact with sick animals [34]. The correlation analysis shows that provinces can explain 43.15% of the heterogeneity of the season subgroup. Summer has different characteristics in the different regions of China’s vast territory, so we further analyzed this result by combining geographical factors. The results showed that the incidence was significantly higher in regions with higher humidity and rainfall, similarly to that in previous studies [35]. In the mean temperature subgroup, the prevalence increased with temperature, but the relationship was not linear as expected. The prevalence peaked at 10–15°C and then declined as temperatures rose. Previous studies have shown that *M*. *bovis* does not easily survive in a hot and dry environment [36], so we speculated that 10–15°C may be the optimum temperature for *M*. *bovis* to survive or to spread, and that a humid environment is conducive to its spread. It is therefore recommended to strengthen epidemic prevention in moist and warm areas to create a healthy living environment for livestock and reduce the incidence of diseases.

In terms of regional subgroups, the prevalence was lowest in Northwestern China and highest in Easthern China (P < 0.05). The geographical factors showed that the longitude range was >110° and the latitude was 20–35°, the highest prevalence was consistent with the regional results. This range belongs to subtropical humid monsoon climate and temperate monsoon climate. It is hot and rainy in summer, mild and humid in winter. The results were basically consistent with the above seasonal and geographical factors. We speculated that warm and humid weather may exacerbate the prevalence of bTB [28,29,31].

Among provinces of China, Shandong had the highest prevalence, while Guangdong and Gansu had the lowest prevalence (P < 0.001). In recent years, a number of modern standardized dairy enterprises that are strong on fundamentals have emerged in Gansu. They have abandoned the extensive model and focused on quantity and quality, so that the dairy farming industry in Gansu has developed well [37]. However, the need for disease prevention and control in cattle in Shandong is still great, and zoonoses occur from time to time due to the low level of cow breeding technology and nonstandard technical operation [38]. In addition, the animal husbandry of Shandong is characterized by early start and high density. Currently, the average carrying capacity per square kilometer of land is 618 standard livestock units, 6.5 times the average in China. Aquaculture pollution and increasing environmental pressure may also contribute to the higher prevalence of bTB [38]. Furthermore, the altitude of Gansu was lower, while that of Shandong was higher, and the prevalence at altitude < 1000 m was significantly higher than other areas. Altitude is inversely related to economy [39]. Transportation is more convenient in economically developed areas, so more animal trade also creates favorable conditions for the spread of bTB [40]. It is worth noting that although 21 provinces or municipalities were included in the study, many provinces had only one or two studies, which could lead to unstable results. It is recommended that all provinces and cities strengthen bTB monitoring to clearly show the regional diversity of bTB in China.

The 100 studies included five methods (ELISA, IFN-γ-ELISA, SIT, SIT& IFN-γ-ELISA, and colloidal gold test). Diverse detection methods usually bring heterogeneity to the meta-analysis of prevalence. Therefore, we used detection methods as a covariate to perform joint analysis with other moderators, and the range of heterogeneity explained by the detection method was only 0–4.95%, which implied that the detection methods had a small influence on each subgroup. The SIT is the standard method for detection of bovine tuberculosis. It involves measuring skin thickness, injecting bovine tuberculin intradermally into the measured area, and measuring any subsequent swelling at the site of injection 72 hours later. SIT (n = 89), which has been approved by the OIE and the European Commission as the main screening tool for bTB, is most frequently used in China [41,42]. The IFN-γ-ELISA uses ELISA to determine gamma interferon in the whole blood sample. It can be tested only by anticoagulation, which is suitable for laboratory diagnosis and early diagnosis of tuberculosis. Cellular immunity and humoral immunity occur in turn when animals are infected with bTB. SIT and the interferon-gamma test are both markers of cellular immunity in the early stage of infection, while ELISA (detection antibody in serum sample) is indicated as a diagnostic method for relatively later infection [43]. Given the prevalence of bTB in different regions, the detection scheme should also be adjusted accordingly. Areas with more severe infection and economic problems should adopt sensitive, scalable, low-cost testing methods to rapidly isolate and remove infected animals to maximize outbreak control. In the regions with stable prevalence, a variety of methods should be adopted for comprehensive testing, which is conducive to improving the accuracy and reducing the incidence of unnecessary slaughter [44]. The colloidal gold test is easy to operate [45]. It should be noted that due to a paucity of studies, the colloidal gold test group and SIT&INF-γ-ELISA group may not fully reflect the true situation of infection; therefore, we have presented these two groups without data pooling in the detection methods subgroup. In conclusion, we believe that the detection of bTB should be tailored to local conditions, and different regions should choose a method based on their actual situation. Without regard to cost and equipment, it is recommended that the SIT, the standard practice recommended by the OIE be used as the main screening experiment and other complementary experiments be used to improve the accuracy of the results. This kind of comprehensive experiment can eliminate infected animals more thoroughly, thereby preventing continuous transmission [46].

We analyzed the research quality and found that most of the articles of medium quality lacked detailed sampling methods and clear sampling time. It is suggested that personnel record relevant information in detail when conducting epidemiological investigation, to provide scientific data and theoretical support for the follow-up study of bTB.

This study’s large sample size and rigorous methods, including comprehensive analysis of moderators, provide a reference for the prevention and control of bTB. However, several limitations may affect this meta-analysis. Firstly, due to the different retrieval strategies of different databases, we used several different retrieval forms and five databases in order to retrieve more qualified studies. Yet there may still be studies that have fallen through the cracks. Secondly, insufficient research on some subgroups (province, season, detection methods, and age) will affect the analysis results to some extent. Thirdly, due to lack of data, we were unable to extract all the potential moderators that are considered important, such as the number of calves produced and cattle breeds [47].

## Conclusion

Our study has shown that bTB is widespread among cattle in China. From our findings about factors affecting prevalence in China, we recommend that countries adapt their own prevention and control policies to local conditions, particularly strengthening the screening of cattle in warm and wet areas, strengthen the breeding of animals and management of animal welfare, and slaughter sick cattle in a timely manner. Carrying out bTB epidemiological investigation in more areas can extend the good foundation that has been laid for the prevention and control of bTB in the future.

## Supporting information

S1 Fig Egger’s test for publication bias. (TIF)

S2 Fig Funnel plot with trim and fill analysis for the publication bias test. (TIF)

S3 Fig Funnel plot with pseudo 95% confidence limit intervals for the examination of publication bias in the feeding mode subgroup. (TIF)

S4 Fig Funnel plot with pseudo 95% confidence limit intervals for the examination of publication bias in the age subgroup. (TIF)

S5 Fig Funnel plot with pseudo 95% confidence limit intervals for the examination of publication bias in the region subgroup. (TIF)

S6 Fig Funnel plot with pseudo 95% confidence limit intervals for the examination of publication bias in the sampling year subgroup. (TIF)

S7 Fig Funnel plot with pseudo 95% confidence limit intervals for the examination of publication bias in the detection methods subgroup. (TIF)

S8 Fig Funnel plot with pseudo 95% confidence limit intervals for the examination of publication bias in the season subgroup. (TIF)

S9 Fig Funnel plot with pseudo 95% confidence limit intervals for the examination of publication bias in the quality level subgroup. (TIF)

S10 Fig Forest plot of the feeding mode subgroup. (TIF)

S11 Fig Forest plot of the age subgroup. (TIF)

S12 Fig Forest plot of the region subgroup. (TIF)

S13 Fig Forest plot of the sampling year subgroup. (TIF)

S14 Fig Forest plot of the detection methods subgroup. (TIF)

S15 Fig Forest plot of the season subgroup. (TIF)

S16 Fig Forest plot of the quality level subgroup. (TIF)

S1 Text Supplementary tables. **Table A.** PRISMA Checklist item. **Table B.** The code in R for this meta-analysis. **Table C.** Egger’s test for publication bias. **Table D.** Included studies and quality scores. **Table E.** Normal distribution test for the normal rate and the different conversion of the normal rate
(DOCX)
