# Supplementary figures and images for: Prevalence of bovine tuberculosis in dairy cattle in China during 2010–2019: A systematic review and meta-analysis
Source: PLoS Negl Trop Dis. 2021 Jun 17;15(6):e0009502. doi: 10.1371/journal.pntd.0009502 (PMC8241035; doi:10.1371/journal.pntd.0009502)

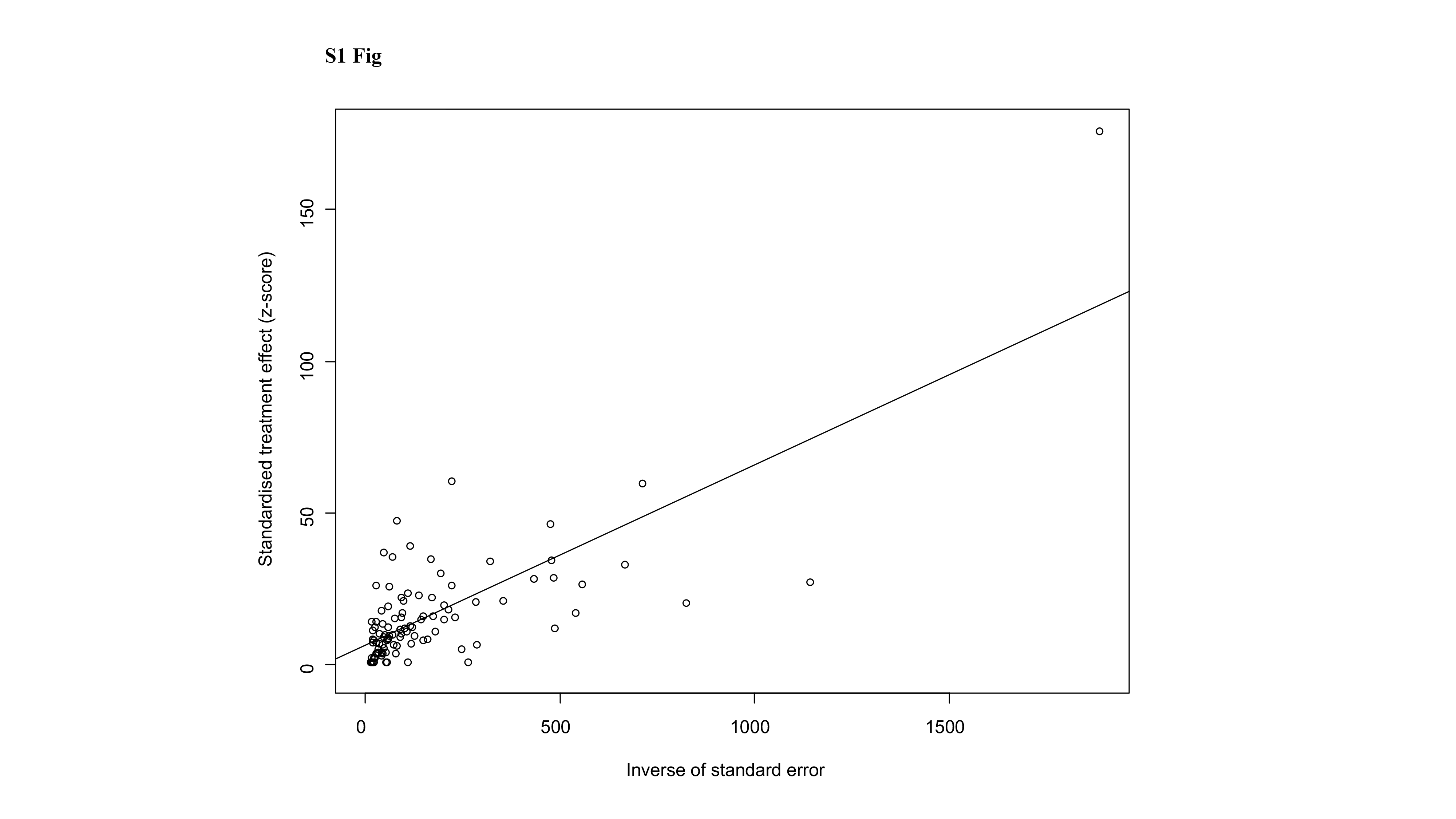

Supplement: S1 Fig — (TIF) [file pntd.0009502.s001.tif]

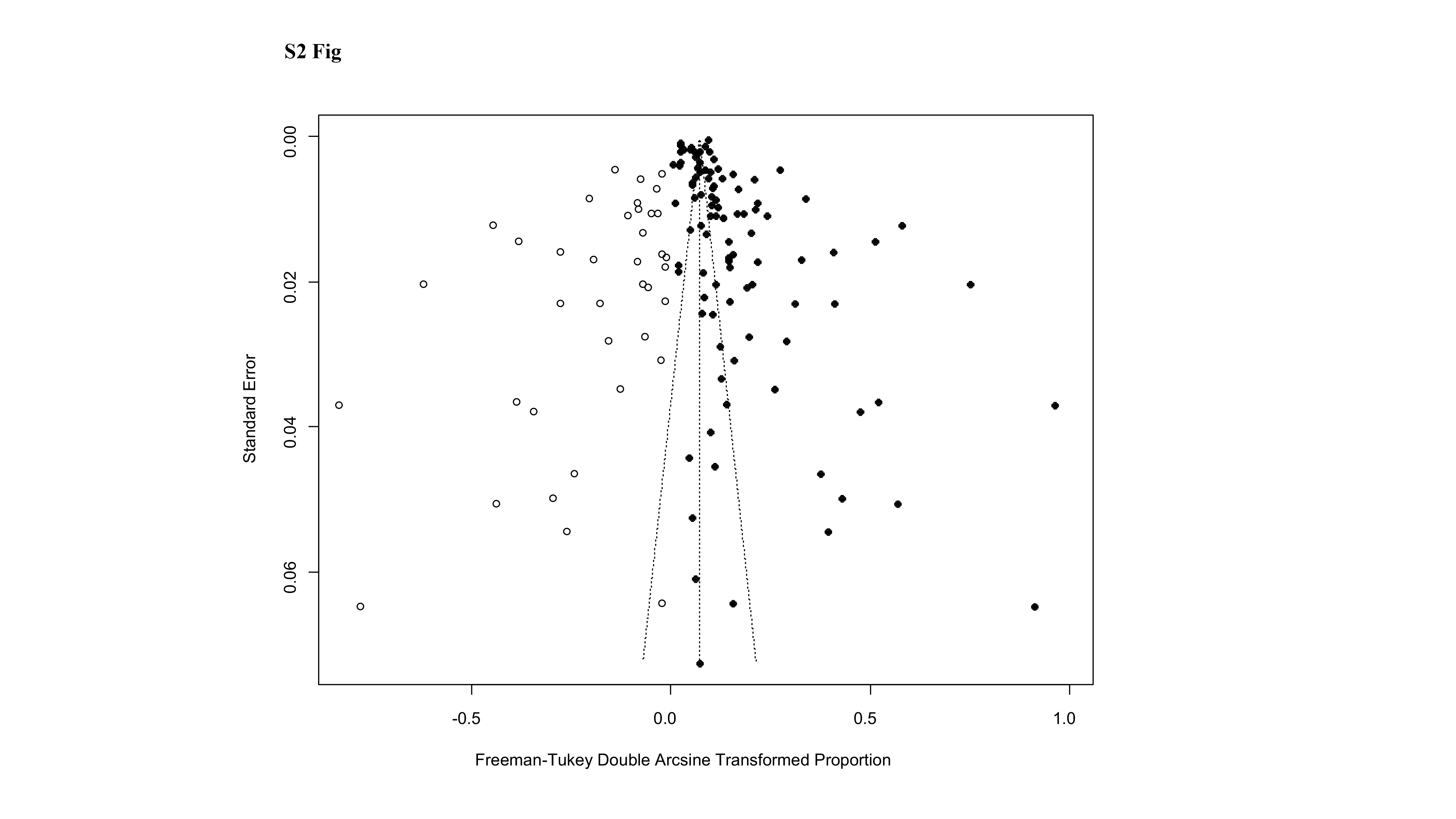

Supplement: S2 Fig — (TIF) [file pntd.0009502.s002.tif]

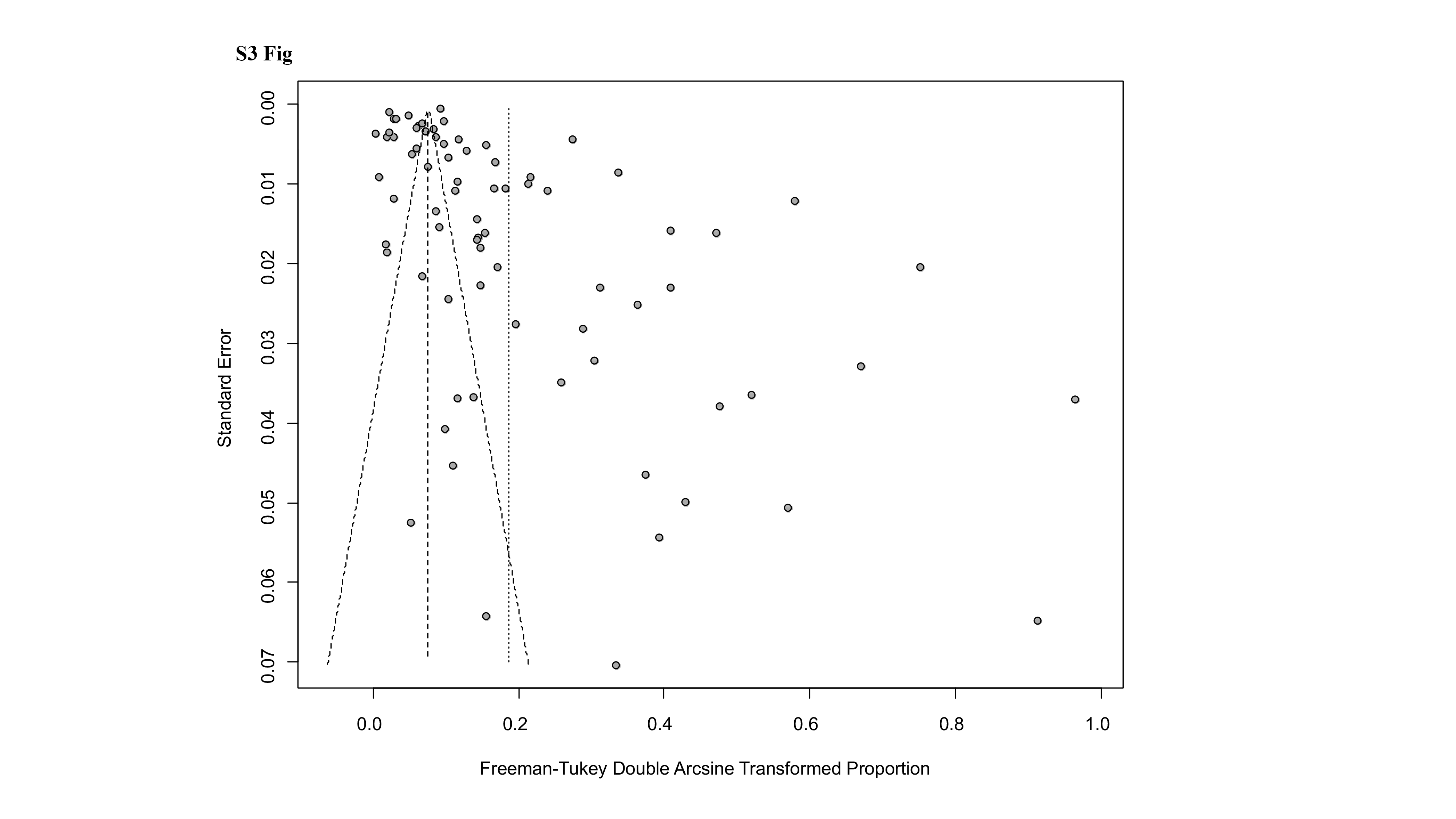

Supplement: S3 Fig — (TIF) [file pntd.0009502.s003.tif]

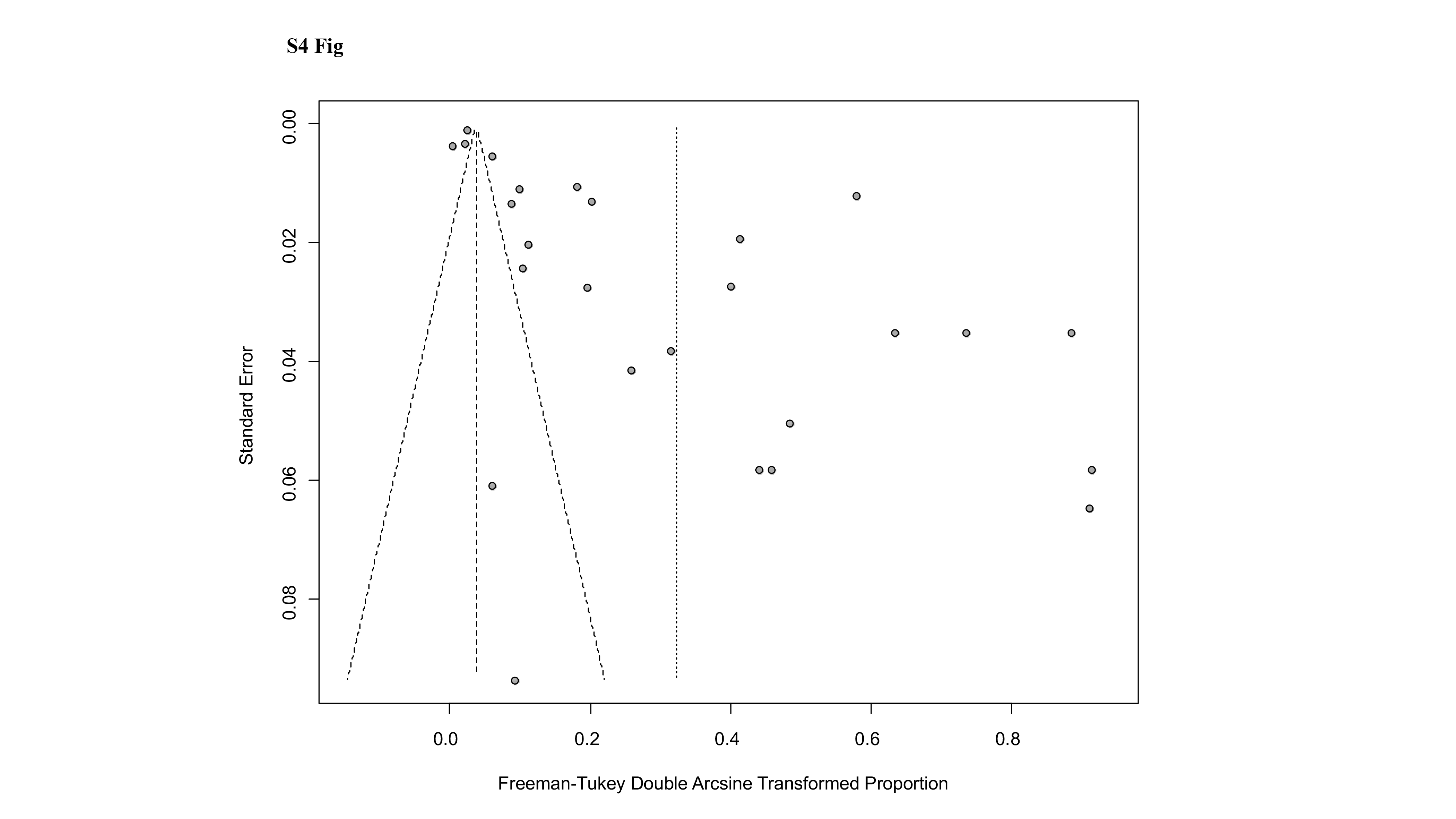

Supplement: S4 Fig — (TIF) [file pntd.0009502.s004.tif]

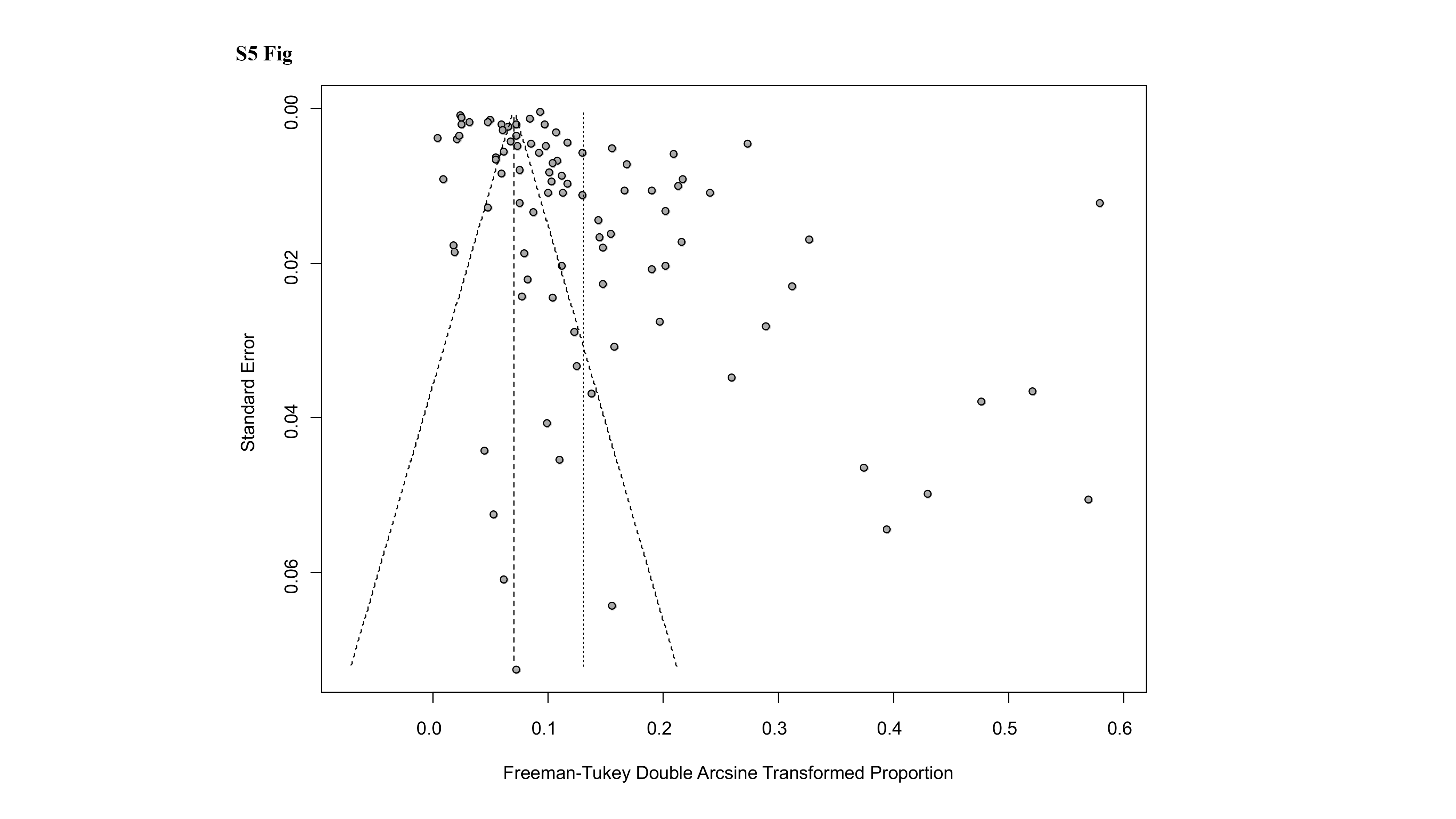

Supplement: S5 Fig — (TIF) [file pntd.0009502.s005.tif]

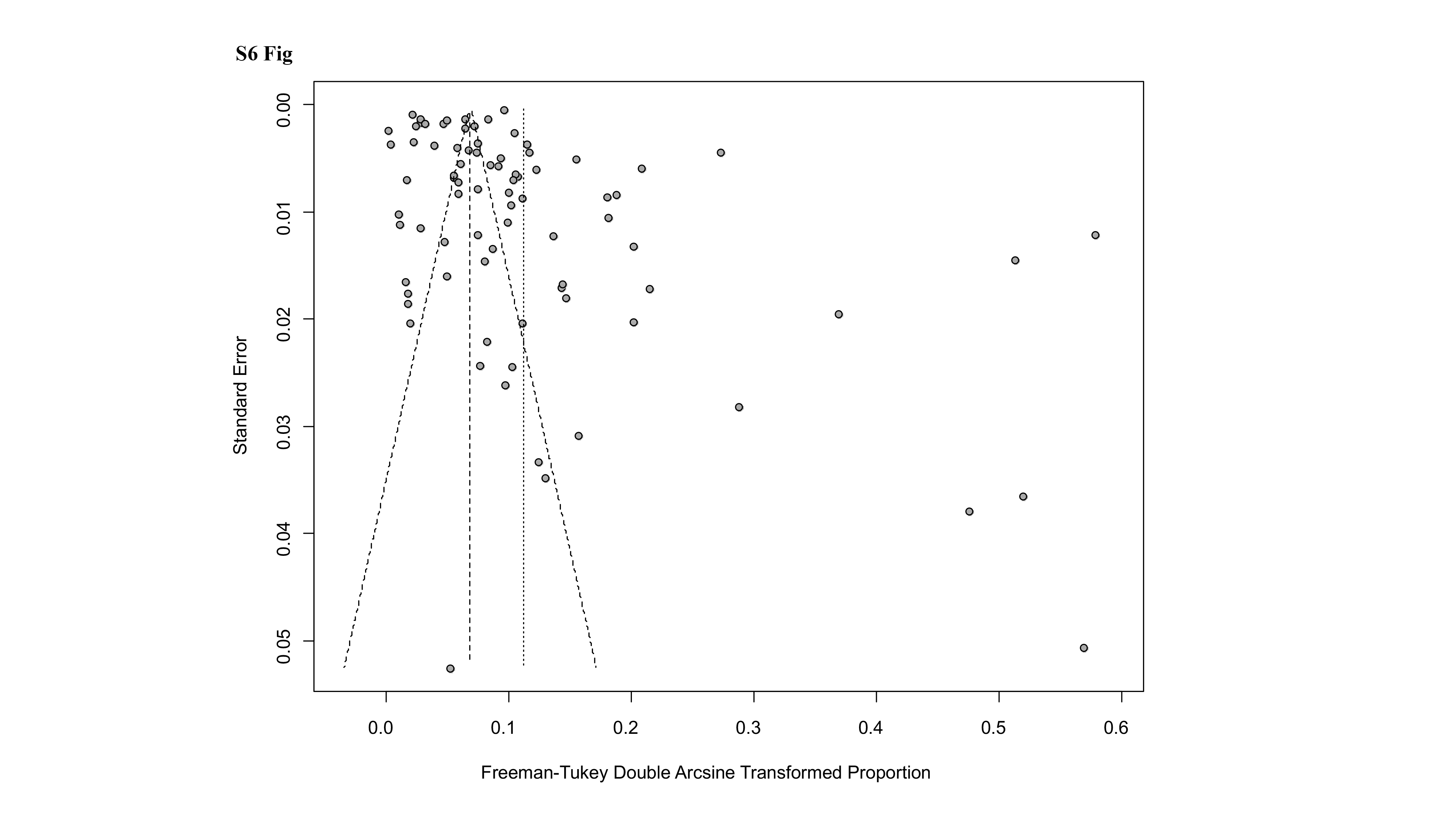

Supplement: S6 Fig — (TIF) [file pntd.0009502.s006.tif]

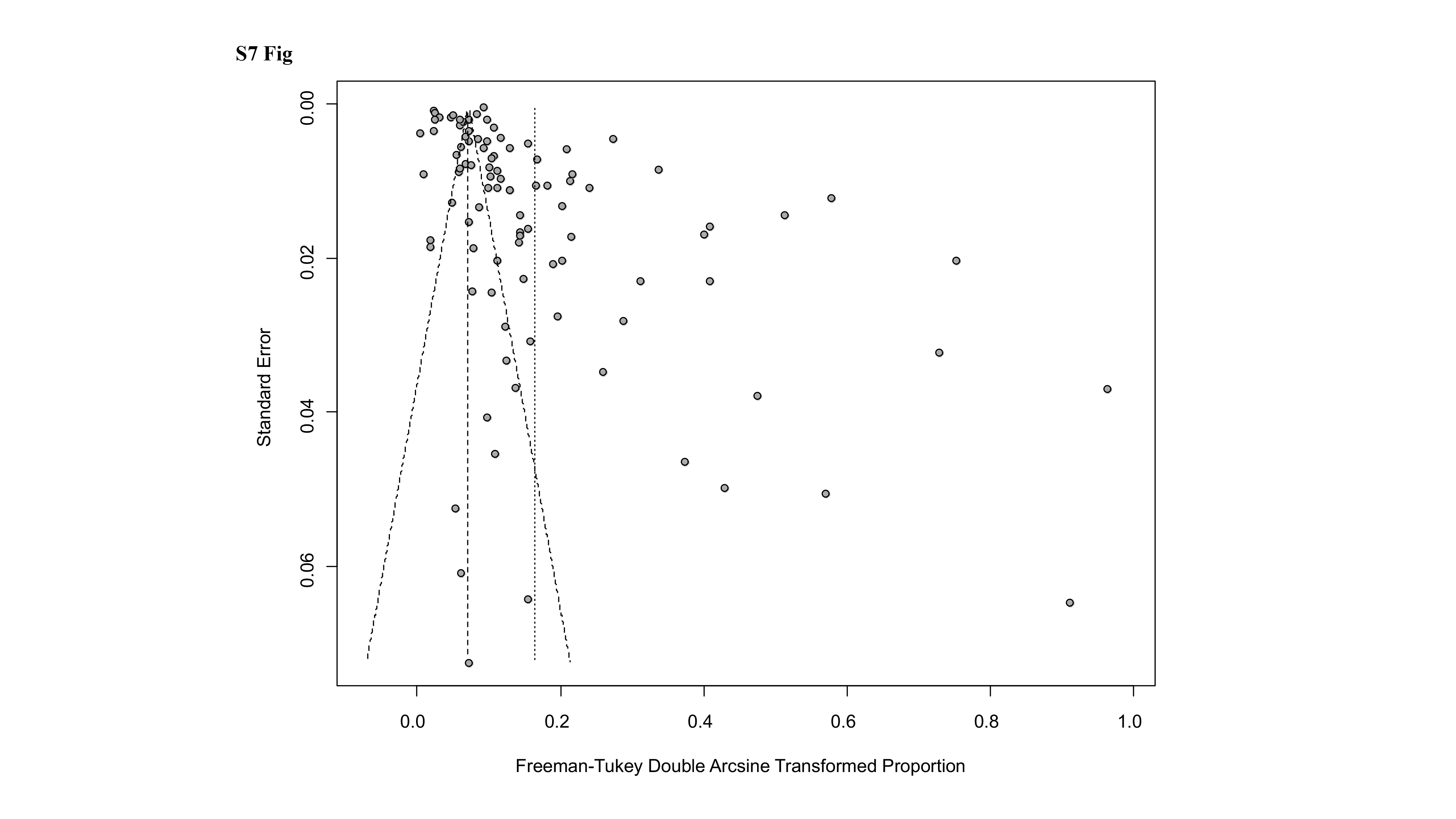

Supplement: S7 Fig — (TIF) [file pntd.0009502.s007.tif]

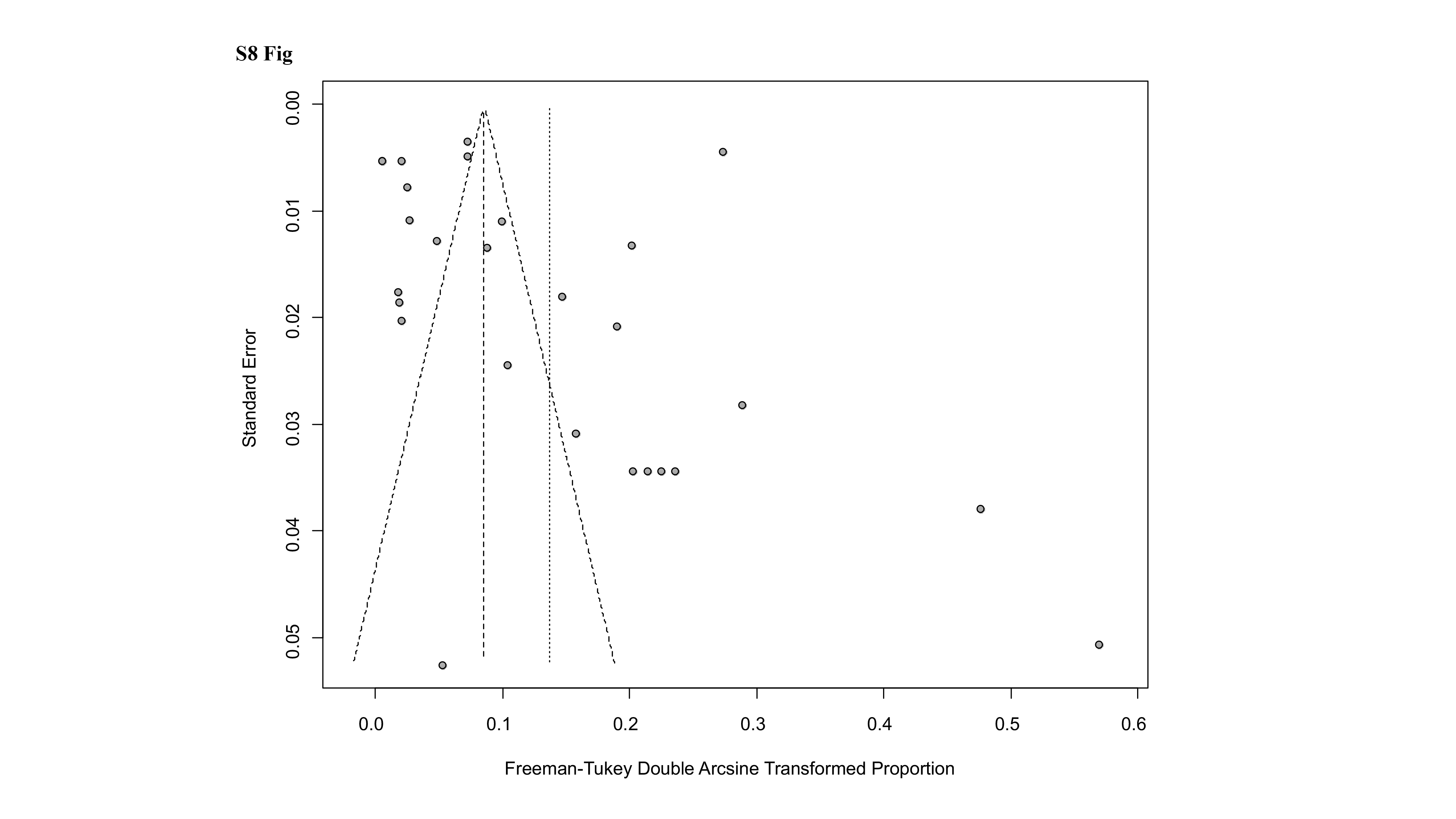

Supplement: S8 Fig — (TIF) [file pntd.0009502.s008.tif]

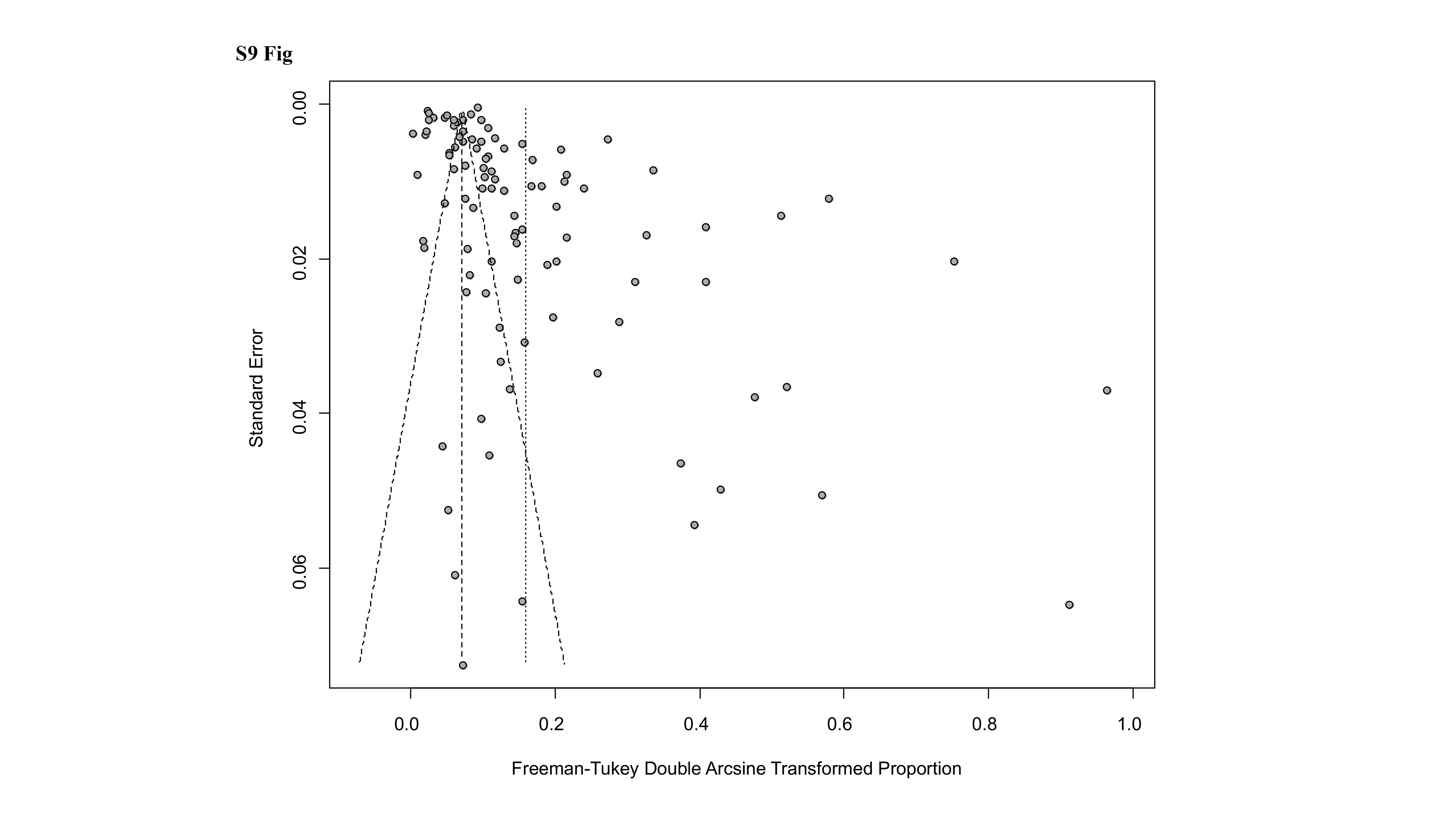

Supplement: S9 Fig — (TIF) [file pntd.0009502.s009.tif]

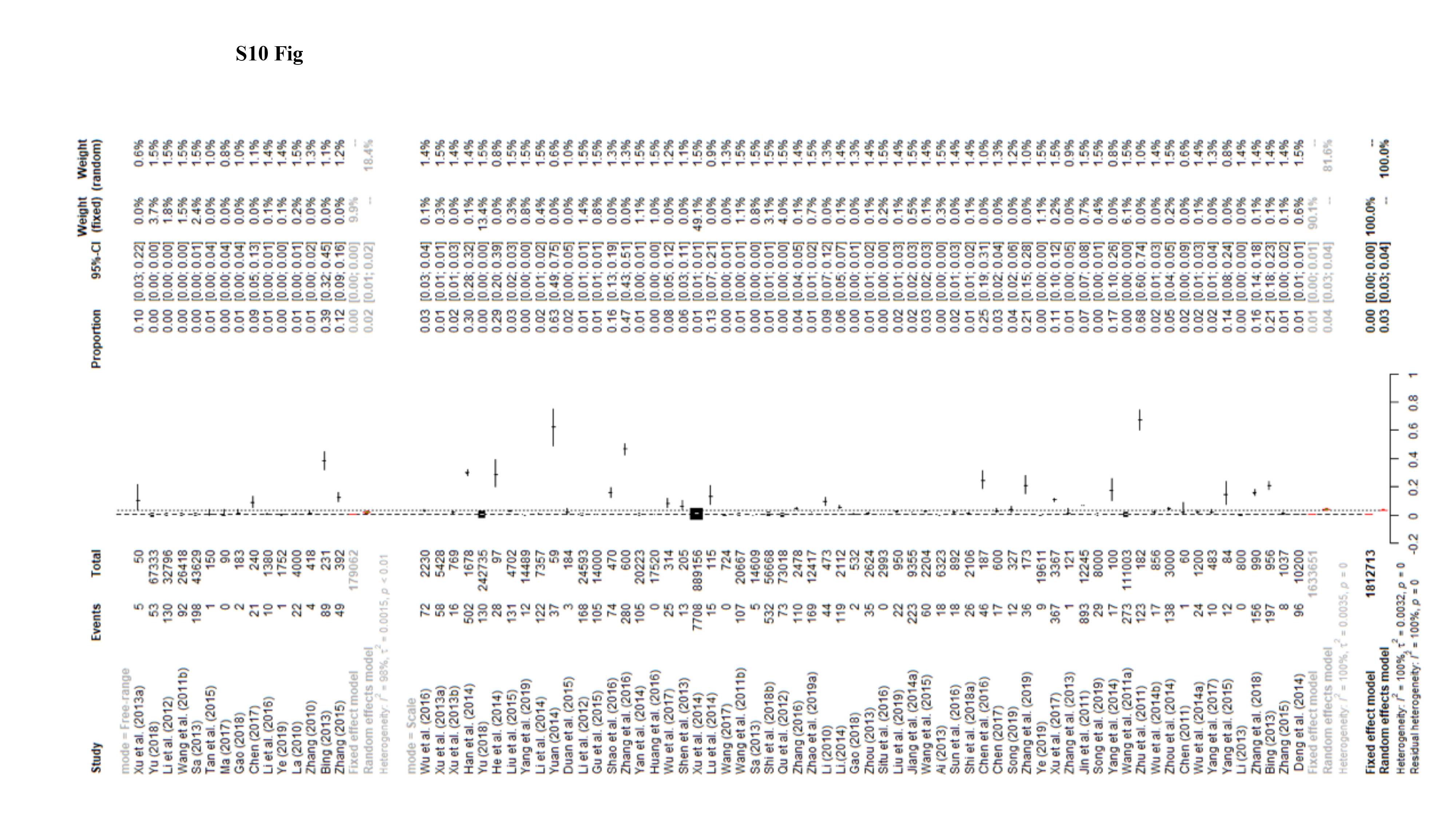

Supplement: S10 Fig — (TIF) [file pntd.0009502.s010.tif]

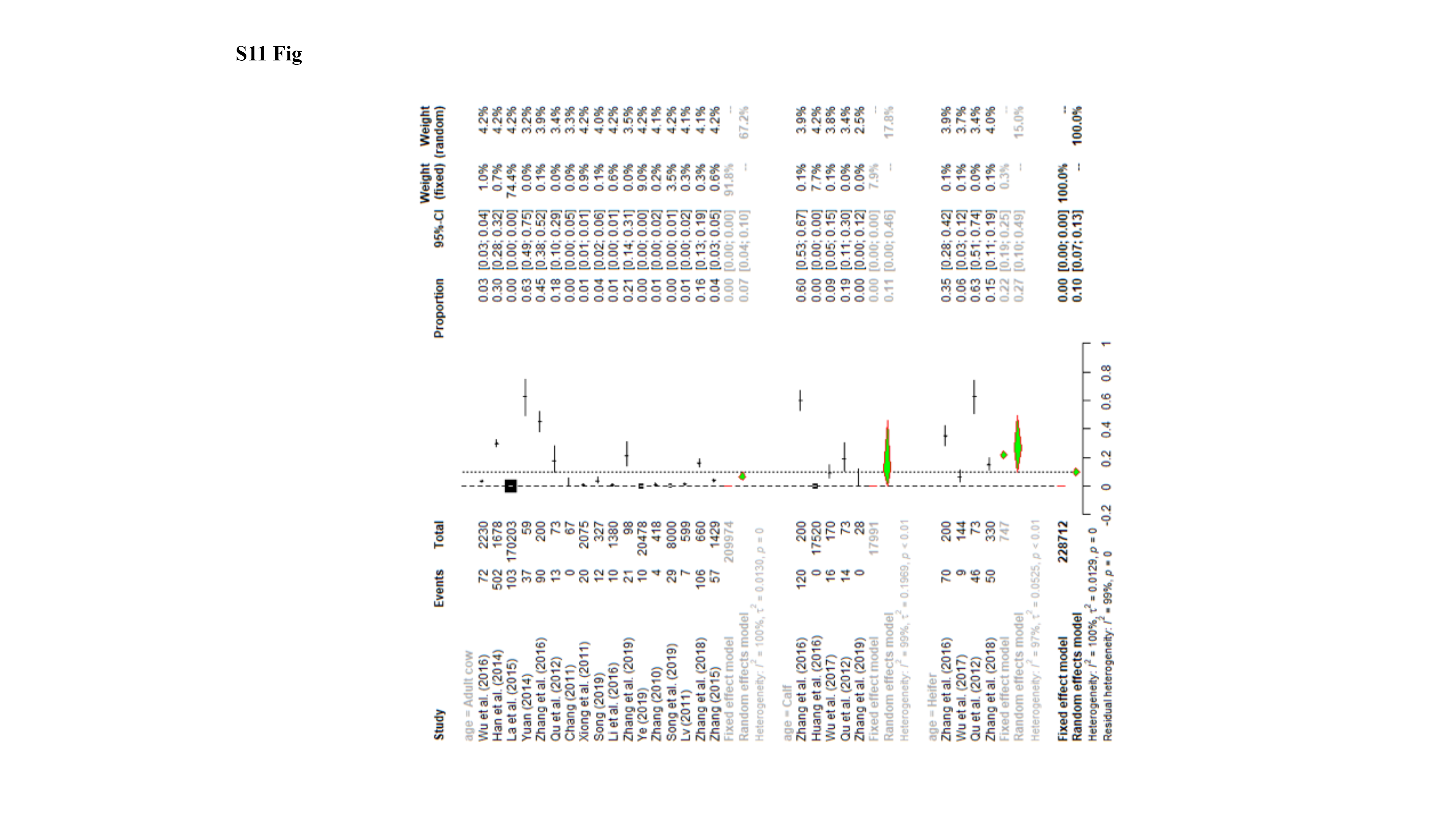

Supplement: S11 Fig — (TIF) [file pntd.0009502.s011.tif]

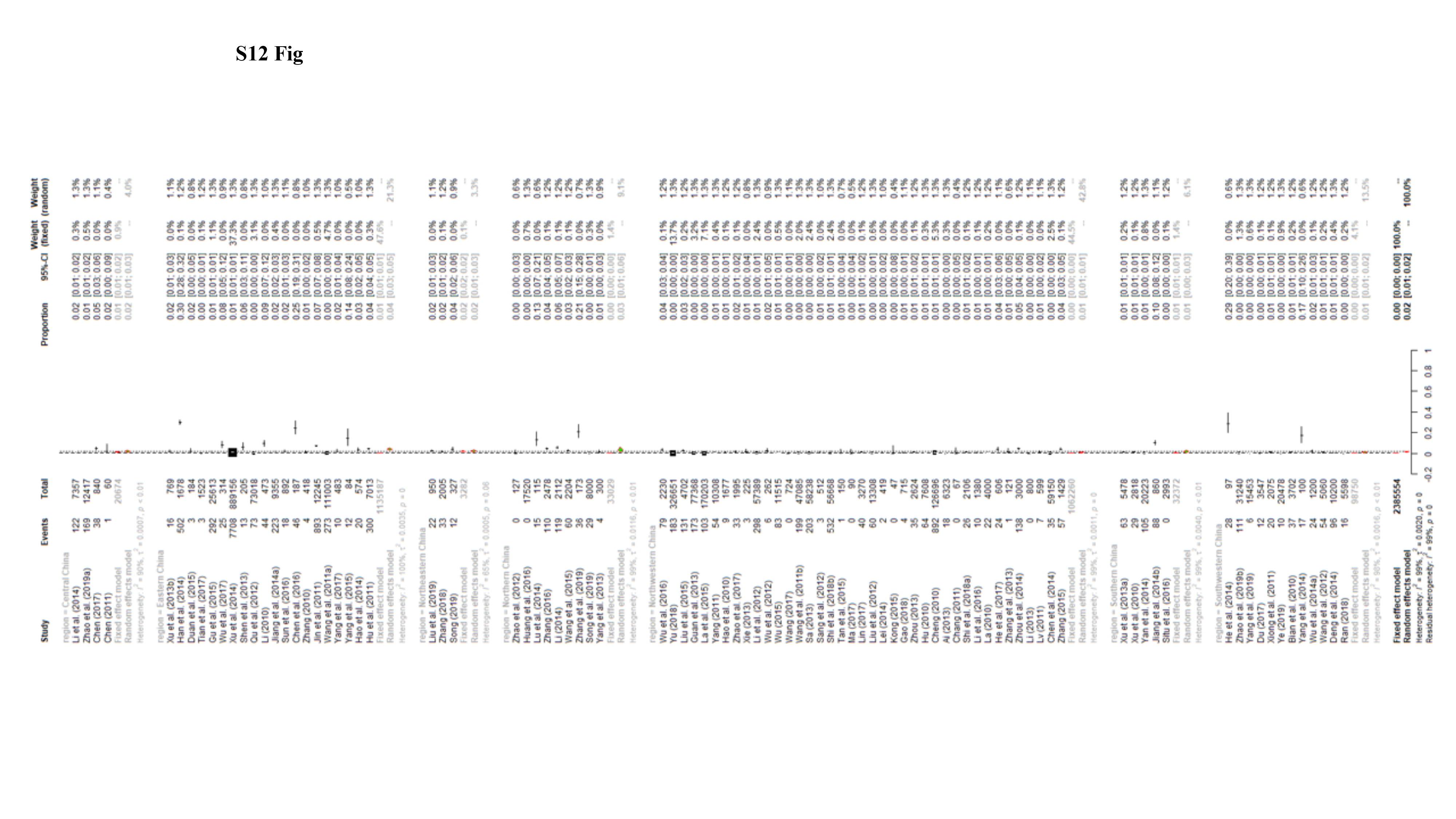

Supplement: S12 Fig — (TIF) [file pntd.0009502.s012.tif]

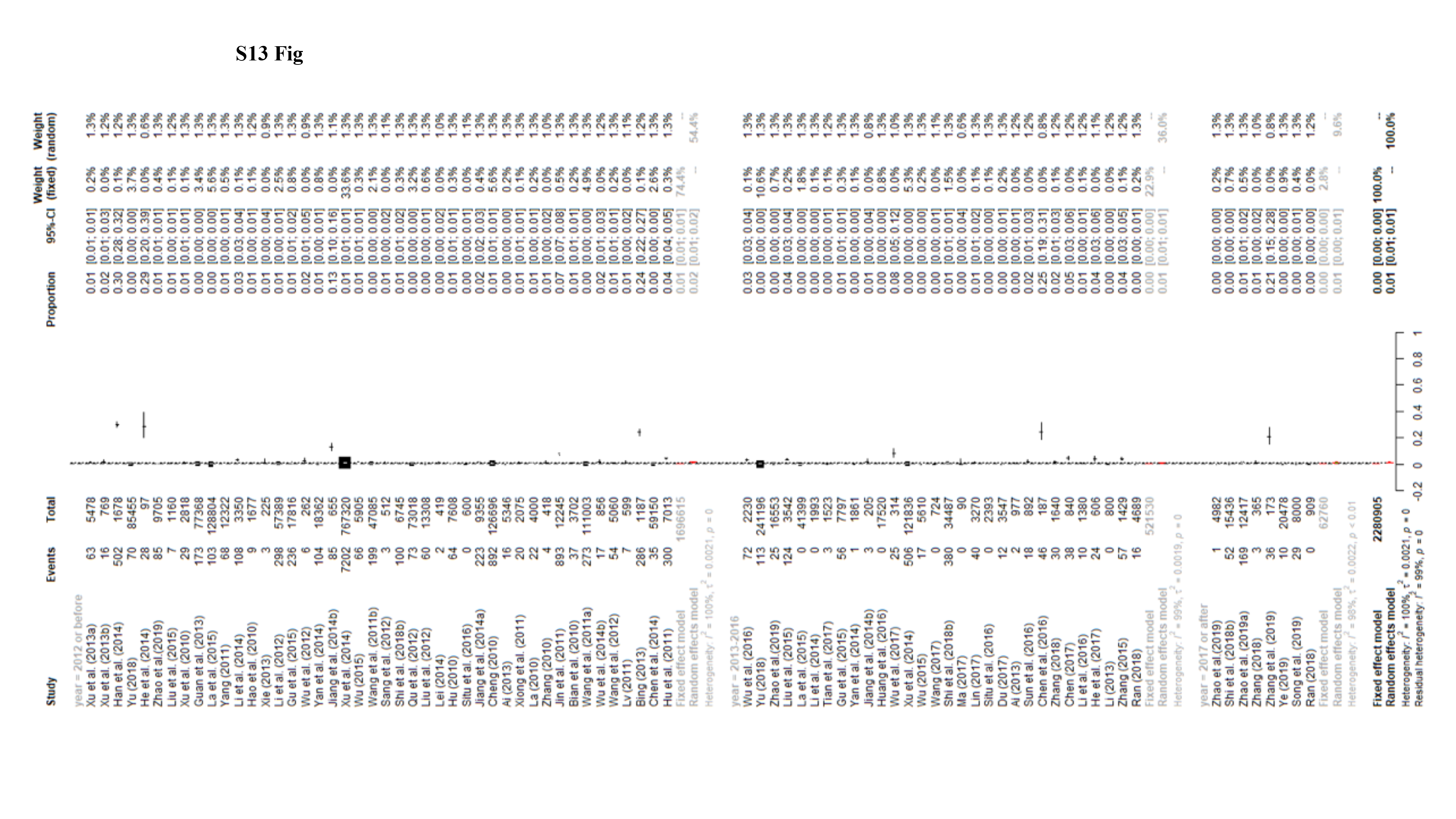

Supplement: S13 Fig — (TIF) [file pntd.0009502.s013.tif]

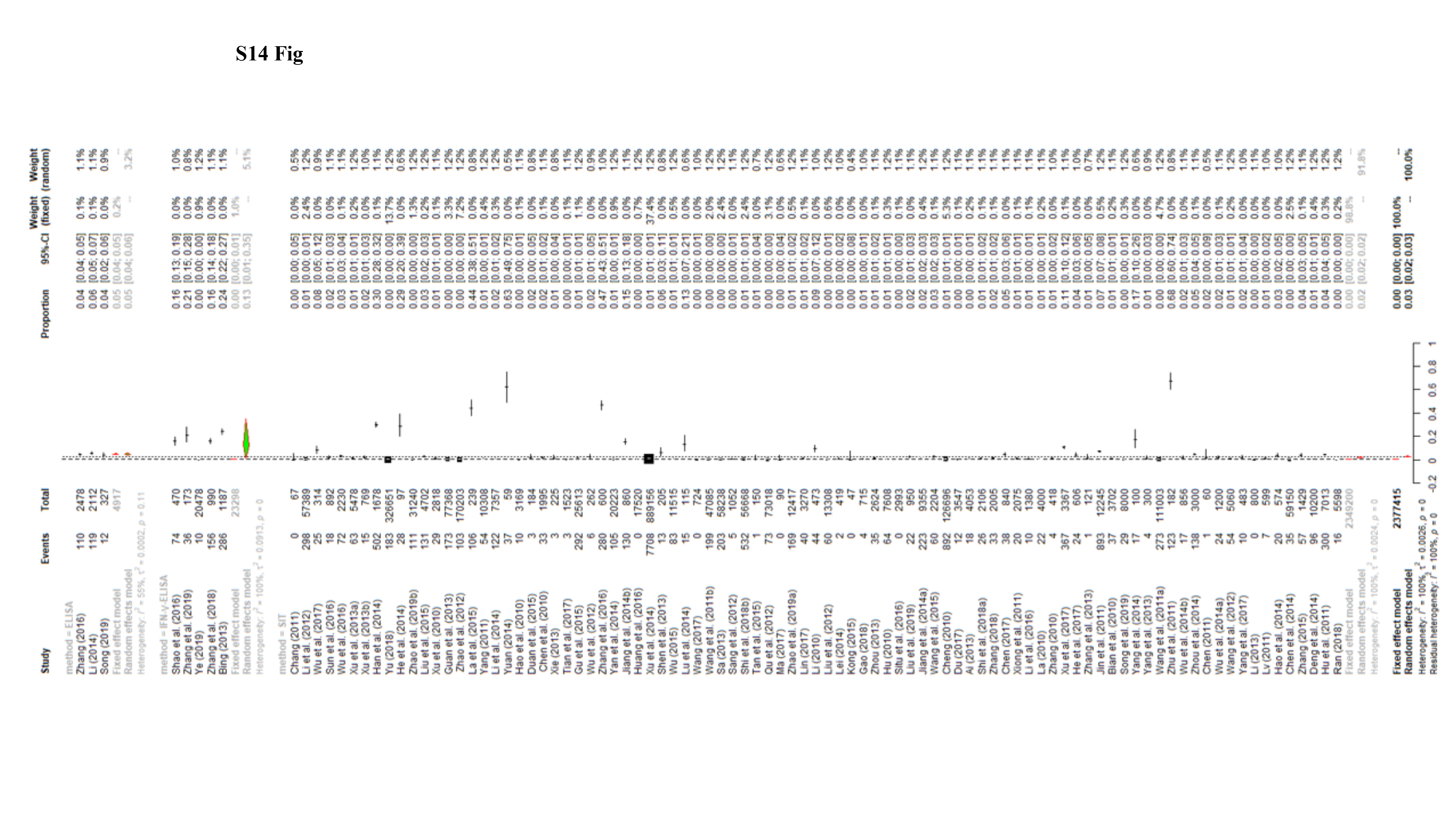

Supplement: S14 Fig — (TIF) [file pntd.0009502.s014.tif]

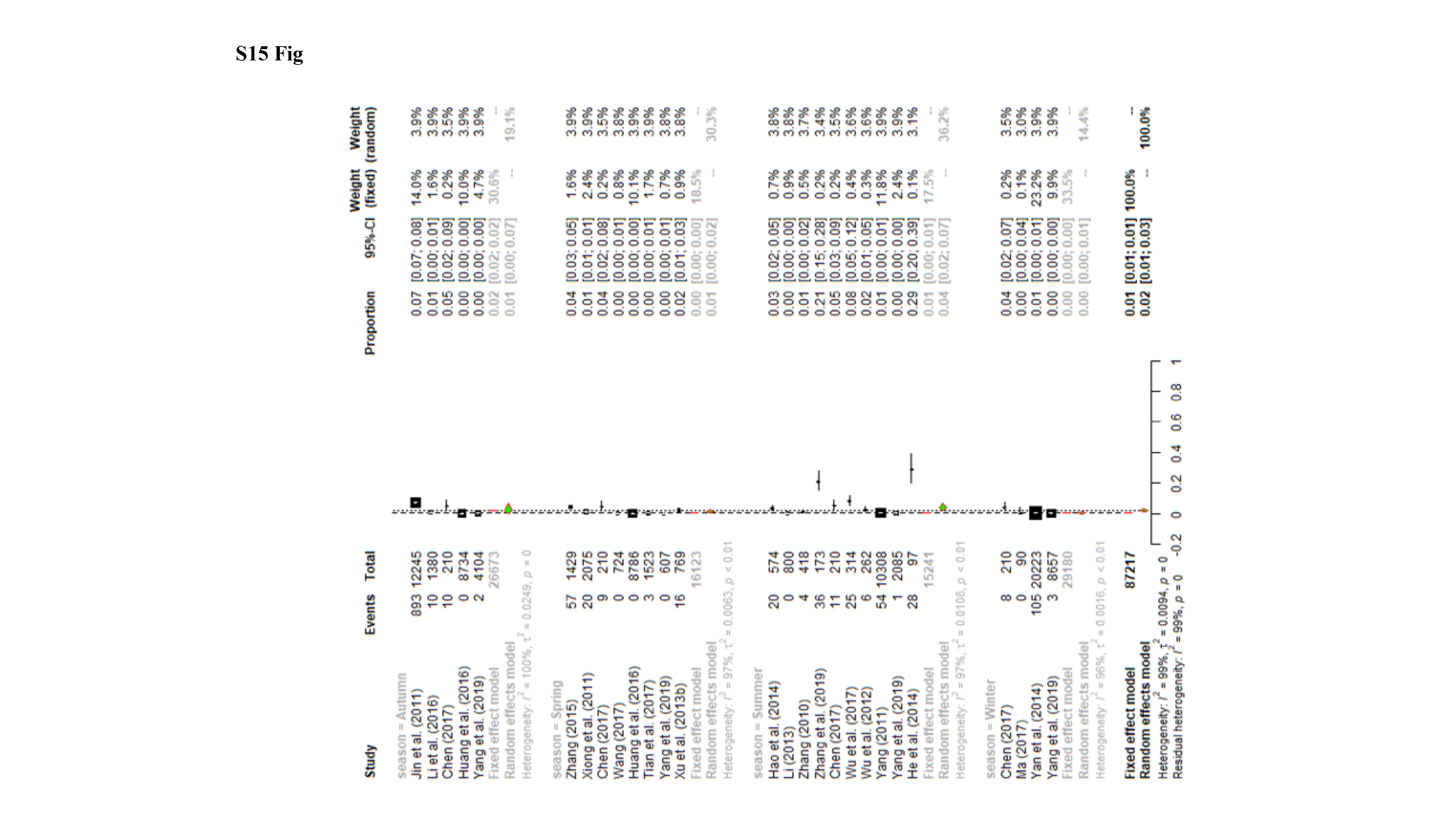

Supplement: S15 Fig — (TIF) [file pntd.0009502.s015.tif]

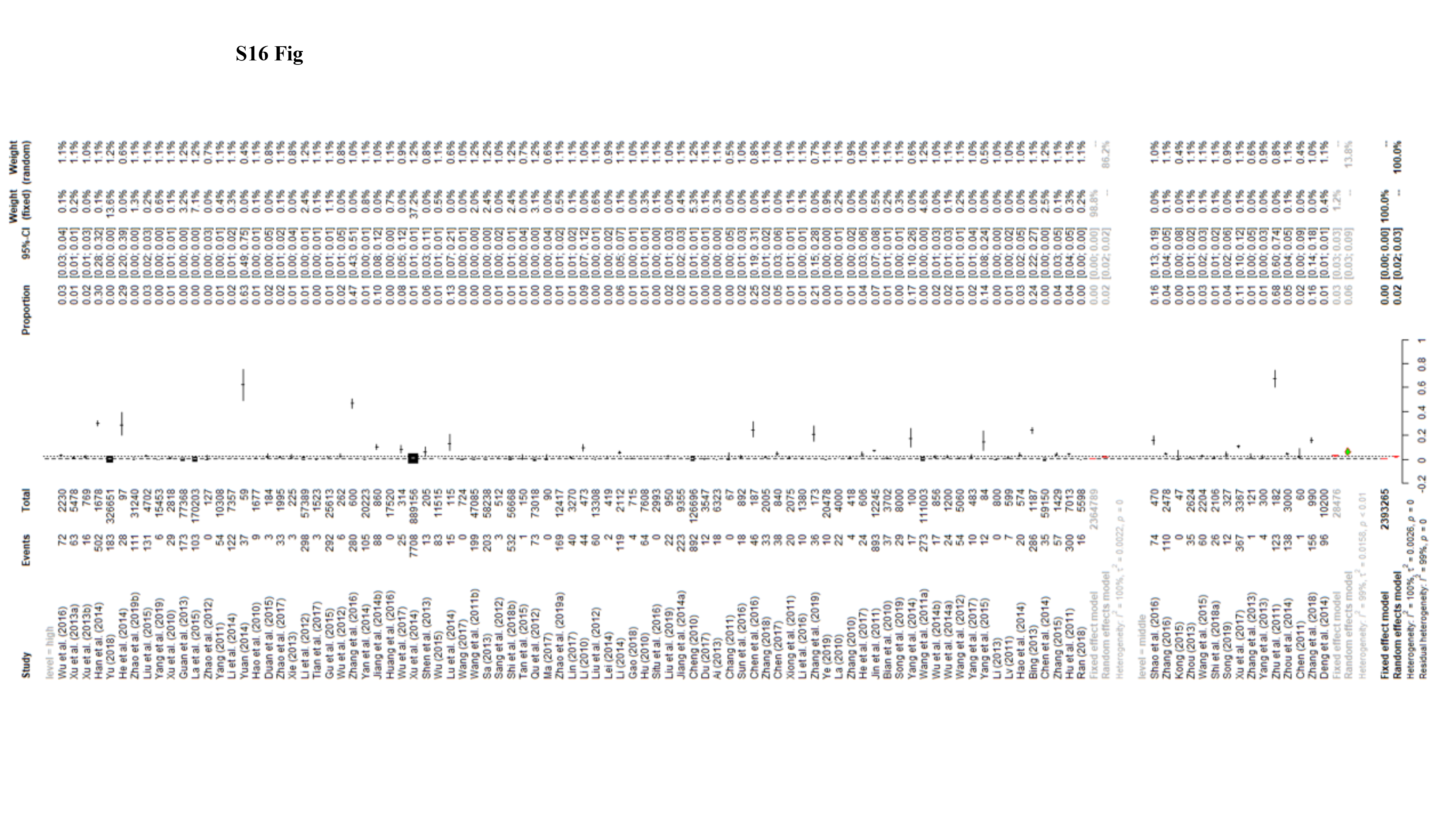

Supplement: S16 Fig — (TIF) [file pntd.0009502.s016.tif]
