# Supplementary material for: Prevalence of bovine tuberculosis in dairy cattle in China during 2010–2019: A systematic review and meta-analysis
Source: PLoS Negl Trop Dis. 2021 Jun 17;15(6):e0009502. doi: 10.1371/journal.pntd.0009502 (PMC8241035; doi:10.1371/journal.pntd.0009502)
Supplement: S1 Text — Table A. PRISMA Checklist item. Table B. The code in R for this meta-analysis. Table C. Egger’s test for publication bias. Table D. Included studies and quality scores. Table E. Normal distribution test for the normal rate and the different conversion of the normal rate (DOCX) [file pntd.0009502.s017.docx]

**Table A** PRISMA Checklist item.

| **Section/topic** | **#** | **Checklist item** | **Reported on page #** |
| --- | --- | --- | --- |
| **TITLE** |  |  |  |
| Title | 1 | Prevalence of bovine tuberculosis in dairy cattle in China during 2010–2019: A systematic review and meta-analysis | 1 |
| **ABSTRACT** |  |  |  |
| Structured summary | 2 | Provide a structured summary including, as applicable: background; objectives; data sources; study eligibility criteria, participants, and interventions; study appraisal and synthesis methods; results; limitations; conclusions and implications of key findings; systematic review registration number. | 2-3 |
| **INTRODUCTION** |  |  |  |
| Rationale | 3 | Describe the rationale for the review in the context of what is already known. | 4-5 |
| Objectives | 4 | Provide an explicit statement of questions being addressed with reference to participants, interventions, comparisons, outcomes, and study design (PICOS). | 4-5 |
| **METHODS** |  |  |  |
| Protocol and registration | 5 | Indicate if a review protocol exists, if and where it can be accessed (e.g., Web address), and, if available, provide registration information including registration number. | 5 |
| Eligibility criteria | 6 | Specify study characteristics (e.g., PICOS, length of follow-up) and report characteristics (e.g., years considered, language, publication status) used as criteria for eligibility, giving rationale. | 5-7 |
| Information sources | 7 | Describe all information sources (e.g., databases with dates of coverage, contact with study authors to identify additional studies) in the search and date last searched. | 5-6 |
| Search | 8 | Present full electronic search strategy for at least one database, including any limits used, such that it could be repeated. | 5-6 |
| Study selection | 9 | State the process for selecting studies (i.e., screening, eligibility, included in systematic review, and, if applicable, included in the meta-analysis). | 6-7 |
| Data collection process | 10 | Describe method of data extraction from reports (e.g., piloted forms, independently, in duplicate) and any processes for obtaining and confirming data from investigators. | 6-7, Table S3 |
| Data items | 11 | List and define all variables for which data were sought (e.g., PICOS, funding sources) and any assumptions and simplifications made. | 6-8 |
| Risk of bias in individual studies | 12 | Describe methods used for assessing risk of bias of individual studies (including specification of whether this was done at the study or outcome level), and how this information is to be used in any data synthesis. | 7-8 |
| Summary measures | 13 | State the principal summary measures (e.g., risk ratio, difference in means). | 7-8 |
| Synthesis of results | 14 | Describe the methods of handling data and combining results of studies, if done, including measures of consistency (e.g., I^2^) for each meta-analysis. | 7-8 |
| Risk of bias across studies | 15 | Specify any assessment of risk of bias that may affect the cumulative evidence (e.g., publication bias, selective reporting within studies). | 7, Figure 3, Figure S1-S9, and Table S4 |
| Additional analyses | 16 | Describe methods of additional analyses (e.g., sensitivity or subgroup analyses, meta-regression), if done, indicating which were pre-specified. | 7-8, Figure 4, Tables 2 and 4. |
| **RESULTS** |  |  |  |
| Study selection | 17 | Give numbers of studies screened, assessed for eligibility, and included in the review, with reasons for exclusions at each stage, ideally with a flow diagram. | 8, Figure 1 |
| Study characteristics | 18 | For each study, present characteristics for which data were extracted (e.g., study size, PICOS, follow-up period) and provide the citations. | Table 1, Table S3 |
| Risk of bias within studies | 19 | Present data on risk of bias of each study and, if available, any outcome level assessment (see item 12). | 15, Figure 3, Figure S1-S9, and Table S4 |
| Results of individual studies | 20 | For all outcomes considered (benefits or harms), present, for each study: (a) simple summary data for each intervention group (b) effect estimates and confidence intervals, ideally with a forest plot. | 8, Figure 2, Figure S10-S16 |
| Synthesis of results | 21 | Present results of each meta-analysis done, including confidence intervals and measures of consistency. | 16-17, Tables 2 and 4 |
| Risk of bias across studies | 22 | Present results of any assessment of risk of bias across studies (see Item 15). | 15, Figure 3, Figure S1-S9, and Table S4 |
| Additional analysis | 23 | Give results of additional analyses, if done (e.g., sensitivity or subgroup analyses, meta-regression [see Item 16]). | 15, Figure 4, Tables 2 and 4 |
| **DISCUSSION** |  |  |  |
| Summary of evidence | 24 | Summarize the main findings including the strength of evidence for each main outcome; consider their relevance to key groups (e.g., healthcare providers, users, and policy makers). | 20-25 |
| Limitations | 25 | Discuss limitations at study and outcome level (e.g., risk of bias), and at review-level (e.g., incomplete retrieval of identified research, reporting bias). | 26 |
| Conclusions | 26 | Provide a general interpretation of the results in the context of other evidence, and implications for future research. | 26 |
| **FUNDING** |  |  |  |
| Funding | 27 | Describe sources of funding for the systematic review and other support (e.g., supply of data); role of founders for the systematic review. | 27 |

*From:* Moher D, Liberati A, Tetzlaff J, Altman DG, The PRISMA Group (2009). Preferred Reporting Items for Systematic Reviews and Meta-Analyses: The PRISMA Statement. PLoS Med 6(6): e1000097. doi:10.1371/journal.pmed1000097

For more information, visit: **www.prisma-statement.org**.

**Table B.** The code in R for this meta-analysis.

| Logarithmic conversion (PNL) | rate<-transform [m1, log=log(event/n)];  shapiro.test(rate$log) |
| --- | --- |
| Logit transformation (PLOGIT) | rate<-transform{m1, logit=log[(event/n)/(1-event/n)]};  shapiro.test(rate$logit) |
| Arcsine transformation (PAS) | rate<-transform{m1, arcsin.size=asin[sqrt(event/(n+1))]};  shapiro.test(rate$arcsin) |
| Double-arcsine transformation (PFT) | rate<-transform{m1,darcsin=0.5*[asin(sqrt(event/(n+1)))+asin((sqrt(event+1)/(n+1)))]};  shapiro.test(rate$darcsin) |
| No transformation (PRAW) | rate<-transform[m1, r= event/n];  shapiro.test(rate$r) |

| Forest plots | forest [meta1, xlim=c(-0.2, 0.8)] |
| --- | --- |
| Funnel chart | funnel (meta1) |
| Egger's test | metabias (meta1, method="linreg") |
| The sensitivity analysis | metainf (meta1, pooled = "random") forest (metainf (meta1, pooled = "random"), xlim=c(0, 0.3)) |
| Subgroup analysis | meta1<-metaprop(event, n, study, data=rate, sm="PLN", incr=0.5, allincr=TRUE, addincr=FALSE, title="", byvar= subgroup title, print.byvar=TRUE) |
| Meta-regression analysis | metareg (meta1, ~covariate title) |

**Table C.** Included studies and quality scores.

| **Study No.** | **Reference ID** | **No. tested** | **No. positive** | **Prevalence** | **Study design** | **Study purpose clear or not** | **Detailed detection method or not** | **Sampled time clearly or not** | **Four or more risk factors or not** | **Score** | **Study Quality** |
| --- | --- | --- | --- | --- | --- | --- | --- | --- | --- | --- | --- |
| 1 | Wu et al. (2016) | 2230 | 72 | 3.23% | Cross sectional | Y | Y | Y | Y | 4 | high quality |
| 2 | Xu et al. (2013a) | 5478 | 63 | 1.15% | Cross sectional | Y | Y | Y | Y | 4 | high quality |
| 3 | Xu et al. (2013b) | 769 | 16 | 2.08% | Cross sectional | Y | Y | Y | Y | 4 | high quality |
| 4 | Han et al. (2014) | 1678 | 502 | 29.92% | Cross sectional | Y | Y | Y | Y | 4 | high quality |
| 5 | Yu (2018) | 326651 | 183 | 0.06% | Cross sectional | Y | N | Y | Y | 3 | high quality |
| 6 | He et al. (2014) | 97 | 28 | 28.87% | Cross sectional | Y | Y | Y | Y | 4 | high quality |
| 7 | Zhao et al. (2019b) | 31240 | 111 | 0.36% | Cross sectional | Y | N | Y | Y | 3 | high quality |
| 8 | Liu et al. (2015) | 4702 | 131 | 2.79% | Cross sectional | Y | Y | Y | Y | 4 | high quality |
| 9 | Yang et al. (2019) | 15453 | 6 | 0.04% | Cross sectional | Y | N | Y | Y | 3 | high quality |
| 10 | Xu et al. (2010) | 2818 | 29 | 1.03% | Cross sectional | Y | Y | Y | N | 3 | high quality |
| 11 | Guan et al. (2013) | 77368 | 173 | 0.22% | Cross sectional | Y | N | Y | Y | 3 | high quality |
| 12 | La et al. (2015) | 170203 | 103 | 0.06% | Cross sectional | Y | N | Y | Y | 3 | high quality |
| 13 | Zhao et al. (2012) | 239 | 106 | 44.35% | Cross sectional | Y | Y | N | Y | 3 | high quality |
| 14 | Yang (2011) | 10308 | 54 | 0.52% | Cross sectional | Y | Y | Y | Y | 4 | high quality |
| 15 | Li et al. (2014) | 7357 | 122 | 1.66% | Cross sectional | Y | Y | Y | Y | 4 | high quality |
| 16 | Yuan (2014) | 59 | 37 | 47.46% | Cross sectional | Y | Y | N | Y | 3 | high quality |
| 17 | Hao et al. (2010) | 1677 | 9 | 0.54% | Cross sectional | Y | Y | Y | Y | 4 | high quality |
| 18 | Duan et al. (2015) | 184 | 3 | 1.63% | Cross sectional | Y | Y | N | Y | 3 | high quality |
| 19 | Zhao et al. (2017) | 1995 | 33 | 1.65% | Cross sectional | Y | N | Y | Y | 3 | high quality |
| 20 | Xie (2013) | 225 | 3 | 1.33% | Cross sectional | Y | Y | Y | Y | 4 | high quality |
| 21 | Li et al. (2012) | 57389 | 298 | 0.52% | Cross sectional | Y | Y | Y | Y | 4 | high quality |
| 22 | Tian et al. (2017) | 1523 | 3 | 0.20% | Cross sectional | Y | Y | Y | Y | 4 | high quality |
| 23 | Gu et al. (2015) | 25613 | 292 | 1.14% | Cross sectional | Y | Y | Y | Y | 4 | high quality |
| 24 | Wu et al. (2012) | 262 | 6 | 2.29% | Cross sectional | Y | N | Y | Y | 3 | high quality |
| 25 | Shao et al. (2016) | 470 | 74 | 15.74% | Cross sectional | Y | Y | N | N | 2 | medium quality |
| 26 | Zhang et al. (2016) | 600 | 280 | 46.67% | Cross sectional | Y | Y | N | Y | 3 | high quality |
| 27 | Yan et al. (2014) | 20223 | 105 | 0.52% | Cross sectional | Y | Y | Y | Y | 4 | high quality |
| 28 | Jiang et al. (2014b) | 860 | 88 | 10.23% | Cross sectional | Y | N | Y | Y | 3 | high quality |
| 29 | Huang et al. (2016) | 17520 | 0 | 0.00% | Cross sectional | Y | Y | Y | Y | 4 | high quality |
| 30 | Wu et al. (2017) | 314 | 25 | 7.96% | Cross sectional | Y | Y | Y | Y | 4 | high quality |
| 31 | Xu et al. (2014) | 889156 | 7708 | 0.87% | Cross sectional | Y | Y | Y | Y | 4 | high quality |
| 32 | Shen et al. (2013) | 205 | 13 | 0.49% | Cross sectional | Y | Y | N | Y | 3 | high quality |
| 33 | Wu (2015) | 11515 | 83 | 0.72% | Cross sectional | Y | Y | Y | Y | 4 | high quality |
| 34 | Lu et al. (2014) | 115 | 15 | 13.04% | Cross sectional | Y | Y | N | Y | 3 | high quality |
| 35 | Wang (2017） | 724 | 0 | 0.00% | Cross sectional | Y | Y | Y | Y | 4 | high quality |
| 36 | Wang et al. (2011b） | 47085 | 199 | 0.42% | Cross sectional | Y | N | Y | Y | 3 | high quality |
| 37 | Sa (2013) | 58238 | 203 | 0.35% | Cross sectional | Y | Y | N | Y | 3 | high quality |
| 38 | Sang et al. (2012) | 512 | 3 | 0.59% | Cross sectional | Y | Y | Y | Y | 4 | high quality |
| 39 | Shi et al. (2018b) | 56668 | 532 | 0.94% | Cross sectional | Y | N | Y | Y | 3 | high quality |
| 40 | Tan et al. (2015) | 150 | 1 | 0.67% | Cross sectional | Y | Y | N | Y | 3 | high quality |
| 41 | Qu et al. (2012) | 73018 | 73 | 0.10% | Cross sectional | Y | Y | Y | Y | 4 | high quality |
| 42 | Ma (2017) | 90 | 0 | 0.00% | Cross sectional | Y | Y | Y | Y | 4 | high quality |
| 43 | Zhang (2016) | 2478 | 110 | 4.44% | Cross sectional | Y | N | N | Y | 2 | medium quality |
| 44 | Zhao et al. (2019a) | 12417 | 169 | 1.36% | Cross sectional | Y | N | Y | Y | 3 | high quality |
| 45 | Lin (2017) | 3270 | 40 | 1.22% | Cross sectional | Y | Y | Y | Y | 4 | high quality |
| 46 | Li (2010) | 473 | 44 | 9.30% | Cross sectional | Y | Y | N | Y | 3 | high quality |
| 47 | Liu et al. (2012) | 13308 | 60 | 0.45% | Cross sectional | Y | Y | Y | Y | 4 | high quality |
| 48 | Lei (2014) | 419 | 2 | 0.48% | Cross sectional | Y | Y | Y | Y | 4 | high quality |
| 49 | Li (2014) | 2112 | 119 | 5.63% | Cross sectional | Y | Y | N | Y | 3 | high quality |
| 50 | Kong (2015) | 47 | 0 | 0.00% | Cross sectional | Y | Y | N | N | 2 | medium quality |
| 51 | Gao (2018) | 715 | 4 | 0.56% | Cross sectional | Y | Y | Y | Y | 4 | high quality |
| 52 | Zhou (2013) | 2624 | 35 | 1.33% | Cross sectional | Y | N | N | Y | 2 | medium quality |
| 53 | Hu (2010) | 7608 | 64 | 0.84% | Cross sectional | Y | N | Y | Y | 3 | high quality |
| 54 | Situ et al. (2016) | 2993 | 0 | 0.00% | Cross sectional | Y | Y | Y | Y | 4 | high quality |
| 55 | Liu et al. (2019) | 950 | 22 | 2.32% | Cross sectional | Y | Y | N | Y | 3 | high quality |
| 56 | Jiang et al. (2014a) | 9355 | 223 | 2.38% | Cross sectional | Y | Y | Y | Y | 4 | high quality |
| 57 | Wang et al. (2015) | 2204 | 60 | 2.72% | Cross sectional | Y | N | N | Y | 2 | medium quality |
| 58 | Cheng (2010) | 126696 | 892 | 0.70% | Cross sectional | Y | N | Y | Y | 3 | high quality |
| 59 | Du (2017) | 3547 | 12 | 0.34% | Cross sectional | Y | Y | Y | Y | 4 | high quality |
| 60 | Ai (2013) | 6323 | 18 | 0.28% | Cross sectional | Y | Y | Y | Y | 4 | high quality |
| 61 | Chang (2011) | 67 | 0 | 0.00% | Cross sectional | Y | Y | N | Y | 3 | high quality |
| 62 | Sun et al. (2016) | 892 | 18 | 2.02% | Cross sectional | Y | Y | Y | Y | 4 | high quality |
| 63 | Shi et al. (2018a) | 2106 | 26 | 1.23% | Cross sectional | Y | N | N | Y | 2 | medium quality |
| 64 | Chen et al. (2016) | 187 | 46 | 24.60% | Cross sectional | Y | Y | Y | Y | 4 | high quality |
| 65 | Zhang (2018) | 2005 | 33 | 1.65% | Cross sectional | Y | N | Y | Y | 3 | high quality |
| 66 | Chen (2017) | 840 | 38 | 4.52% | Cross sectional | Y | Y | Y | Y | 4 | high quality |
| 67 | Xiong et al. (2011) | 2075 | 20 | 0.96% | Cross sectional | Y | Y | Y | Y | 4 | high quality |
| 68 | Song (2019) | 327 | 12 | 3.67% | Cross sectional | Y | N | N | Y | 2 | medium quality |
| 69 | Li et al. (2016) | 1380 | 10 | 0.72% | Cross sectional | Y | Y | Y | Y | 4 | high quality |
| 70 | Zhang et al. (2019) | 173 | 36 | 20.81% | Cross sectional | Y | Y | Y | Y | 4 | high quality |
| 71 | Ye (2019) | 20478 | 10 | 0.05% | Cross sectional | Y | Y | Y | Y | 4 | high quality |
| 72 | La (2010) | 4000 | 22 | 0.55% | Cross sectional | Y | N | Y | Y | 3 | high quality |
| 73 | Zhang (2010) | 418 | 4 | 0.96% | Cross sectional | Y | Y | Y | Y | 4 | high quality |
| 74 | Xu et al. (2017) | 3367 | 367 | 5.76% | Cross sectional | Y | Y | N | N | 2 | medium quality |
| 75 | He et al (2017) | 606 | 24 | 3.96% | Cross sectional | Y | N | Y | Y | 3 | high quality |
| 76 | Zhang et al. (2013) | 121 | 1 | 0.83% | Cross sectional | Y | N | N | Y | 2 | medium quality |
| 77 | Jin et al. (2011) | 12245 | 893 | 7.29% | Cross sectional | Y | Y | Y | Y | 4 | high quality |
| 78 | Bian et al. (2010) | 3702 | 37 | 1.00% | Cross sectional | N | Y | Y | Y | 3 | high quality |
| 79 | Song et al. (2019) | 8000 | 29 | 0.36% | Cross sectional | Y | Y | Y | Y | 4 | high quality |
| 80 | Yang et al. (2014) | 100 | 17 | 17.00% | Cross sectional | Y | Y | N | Y | 3 | high quality |
| 81 | Yang et al. (2013) | 300 | 4 | 1.00% | Cross sectional | Y | Y | N | N | 2 | medium quality |
| 82 | Wang et al. (2011a) | 111003 | 273 | 0.25% | Cross sectional | Y | N | Y | Y | 3 | high quality |
| 83 | Zhu et al. (2011) | 182 | 123 | 21.98% | Cross sectional | Y | Y | N | N | 2 | medium quality |
| 84 | Wu et al. (2014b) | 856 | 17 | 1.99% | Cross sectional | Y | Y | Y | Y | 4 | high quality |
| 85 | Zhou et al. (2014) | 3000 | 138 | 4.60% | Cross sectional | Y | N | N | Y | 2 | medium quality |
| 86 | Chen (2011) | 60 | 1 | 1.67% | Cross sectional | Y | N | N | Y | 2 | medium quality |
| 87 | Wu et al. (2014a) | 1200 | 24 | 2.00% | Cross sectional | Y | Y | N | Y | 3 | high quality |
| 88 | Wang et al. (2012) | 5060 | 54 | 1.07% | Cross sectional | Y | Y | Y | Y | 4 | high quality |
| 89 | Yang et al. (2017) | 483 | 10 | 2.07% | Cross sectional | Y | Y | N | Y | 3 | high quality |
| 90 | Yang et al. (2015) | 84 | 12 | 14.29% | Cross sectional | Y | Y | N | Y | 3 | high quality |
| 91 | Li (2013) | 800 | 0 | 0.00% | Cross sectional | Y | N | Y | Y | 3 | high quality |
| 92 | Lv (2011) | 599 | 7 | 1.17% | Cross sectional | Y | N | Y | Y | 3 | high quality |
| 93 | Hao et al. (2014) | 574 | 20 | 3.48% | Cross sectional | Y | Y | Y | Y | 4 | high quality |
| 94 | Zhang et al. (2018) | 990 | 156 | 15.76% | Cross sectional | Y | Y | N | N | 2 | medium quality |
| 95 | Bing (2013) | 1187 | 286 | 24.09% | Cross sectional | Y | Y | Y | Y | 4 | high quality |
| 96 | Chen et al. (2014) | 59150 | 35 | 0.06% | Cross sectional | Y | N | Y | Y | 3 | high quality |
| 97 | Zhang (2015) | 1429 | 57 | 3.99% | Cross sectional | Y | N | Y | Y | 3 | high quality |
| 98 | Deng et al. (2014) | 10200 | 96 | 0.94% | Cross sectional | Y | N | N | Y | 2 | medium quality |
| 99 | Hu et al. (2011) | 7013 | 300 | 4.28% | Cross sectional | Y | N | Y | Y | 3 | high quality |
| 100 | Ran (2018) | 5598 | 16 | 0.29% | Cross sectional | Y | Y | Y | Y | 4 | high quality |

Y*: Yes; N*: No.

**References**

1. Wu, Y. W., Wang, X. L., Zhang, Y. L., Wang, Y. M., Li, Z. H., & Zhang, X. J. (2016). Applications of IFN-γ ELISA for Epidemiological Survey of Bovine Tuberculosis. *China Anim Health Inspection, 33*(09), 78-80. (In Chinese)
2. Xu, X. K., Xiong, Y., Wei, D. Y., Huang, X. W., Lan, J., Huang, S. B., . . . Liu, Q. (2013). Epidemiological survey of bovine tuberculosis in Guangxi using interferon gamma ELISA detection. *J South Agr, 44*(04), 667-670. (In Chinese)
3. Xu, J. R., Chen, J., Zhu, G. Q., Chen, X., Zhang, X. J., & Da, J. S. (2013). Clinical application of γ-interferon release test and intradermal allergy test in the detection of bovine tuberculosis. *Heilongjiang Anim Sci Vet Med, 56*(11), 118-121. (In Chinese)
4. Han, M., Zhang, L., Wang, J. L., Ding, J. B., Liu, L. X., Gu, Z.Y., . . . Yang, H. J. (2014). Epidemiological Investigation of Large-scale Dairy Farm on Tuberculosis and Paratuberculosis in Shandong Province. *Chin Agr Sci Bull, 30*(14), 10-13. (In Chinese)
5. Yu, Q. L. (2018). Results of monitoring of tuberculosis in dairy cows in Gansu Province from 2011 to 2016 Analysis and prevention and control recommendations. *China Dairy Cattle, 36*(01), 27-29. (In Chinese)
6. He, J., Pu, H. J., Lu, Y. F., Fan, X., & Pu, G. Y. (2014). Investigation of Dairy Cow Tuberculosis in Kaiyuan City. *China Anim Livest Vet Digest, 30*(12), 85. (In Chinese)
7. Zhang, H. T., Yang, J., Wei, J. W., Du, P. W., & Duan, B. F.(2019). Surveillance and Analysis of Tuberculosis of Dairy Cows in Eryuan County of Yunnan Province during 2012-2018. *J Anhui Agr Sci,* *47*(18), 105-107. (In Chinese)
8. Liu, X. P., Wang, Z. S., Ma, C. B., Zhou, G. Z., Li, L., Wu, T., . . . Zeng, S. (2015). Investigation and thinking on the epidemic situation of tuberculosis in a large-scale dairy farm. *China Cattle Sci, 41*(4), 49-51. (In Chinese)
9. Yang, A. G., Lu, Z. P., Hou, W., Mo, Q., Zhou, M. Z., Yin, J., . . . Yang, Z. C. (2019). Epidemiological survey of tuberculosis in dairy cows in the main dairy cattle breeding areas in Sichuan Province from 2016 to 2017. *Heilongjiang Anim Sci Vet Med, 62*(10), 93-95. (In Chinese)
10. Xu, X. K., Huang, X. W., Lan, J., Huang, S. B., Xiong, Y., Liu, Q., Wei, Z. J., . . . Huang, Y. H. (2010). Comparison of two methods in the diagnosis of bovine tuberculosis. *Anim Livest Vet Med, 42*(02), 29-33. (In Chinese)
11. Guan, Q., Ma, Y. L., Xiao., K. T., & Shi, Y. X. (2013). Epidemiological investigation and surveillance analysis of designated tuberculosis in Xinjiang dairy cows from 2010 to 2012. *Xinjiang Anim Livest, 29*(4), 29-30. (In Chinese)
12. La, H., Wang, S. X., Wang, X. Y., Ma, R. L., Fu, Y. J., Wang, Z. F., . . . Wang, X. Z. (2015). Surveillance and epidemic trend analysis of bovine tuberculosis in Qinghai Province in 2004. *Heilongjiang Anim Sci Vet Med, 58*(10), 91-93. (In Chinese)
13. Zhao, J. Y., Jiang, Y. M., Wei, H. T., Liu, X. D., Xu, F. R., Guan, J. M., . . . Xu, J. P. (2012). BOVIGAM^®^ Mycobacterium bovis γ-interferon detection method and comparison test of intradermal allergy. The Twelfth Symposium on Zoonoses and the Proceedings of the Sixth Fourteenth Teaching Committee of the Chinese Society of Animal Husbandry and Veterinary Medicine. (In Chinese)
14. Yang, L. C. (2011). Surveillance and control measures of brucellosis and tuberculosis in Minhe County. *China Dairy Cattle, 29*(01), 59-62. (In Chinese)
15. Li, X. S., Shen, C., Xu, Y. X., & Yang, F. L. (2014). Investigation and control measures of milk cow tuberculosis in Huangzhou District of Huanggang City. *Heilongjiang Anim Sci Vet Med. 57*(11), 78-79. (In Chinese)
16. Yuan, Q. F. (2014). IFN-γ in vitro release method for detection of bovine tuberculosis. *China Anim Health, 16*(7), 32-35. (In Chinese)
17. Hao, C. C., Shi, J. H., & Yao, H. R. (2010). Investigation on Dairy Cow Tuberculosis in Hualong County. *Modern Agr Sci Technol, 39*(09), (In Chinese)
18. Duan, Z. T., & Zhuang, J. Y. (2015). Application test of SICT and ELISA in the detection of tuberculosis in dairy cows. *Zhejiang J Anim Sci Vet Med, 61*(5), 4-5. (In Chinese)
19. Zhao, B., Zhao, H. T., Zhao, Y. P., Yan, H. T., Lan, Z. R., & Zhang, C. L. (2017). A Cross-sectional Investigation on Prevalence of Tuberculosis in Large-scale Dairy Farms in a County of Shaanxi Province. *China Anim Health Inspect, 36*(3). (In Chinese)
20. Xie, R. Z. M. (2013). Serological Detection of Tuberculosis in Dairy Cows in Gaba Songdu Town, Tongde County. *Hubei J Anim Vet Sci, 34*(10), 19-20. (In Chinese)
21. Li, A. Q., Zhao, J. G., Hu, D. J., Chen, B., & Wang, L. H. (2012). Epidemiological investigation and prevention of tuberculosis in dairy cows in Urumqi. *China Anim Health Inspect, 29*(10), 52-53, 56. (In Chinese)
22. Tian, G. Z., Tian, X. F., & Zhou, S. L. (2017). Epidemiological investigation of tuberculosis in dairy cows in Mudan District of Heze City. *Shandong J Anim Sci Vet Med, 38*(01), 54. (In Chinese)
23. Gu, S. S., & Fan, F. (2017). Epidemiological investigation of tuberculosis in dairy cows in Wuxi City in recent years. *Chin Livest Poult Breed, 11*(03), 3-5. (In Chinese)
24. Wu, W. F. E. (2012). Epidemiological investigation of tuberculosis in dairy cows in Hejing County. *Xinjiang Anim Livest, 28*(S1):24-25. (In Chinese)
25. Shao, H. X., Zhao, W., Huang, J., Qian, K., Ye, J. Q., & Qin, A. J. (2016). Comparison of the effects of different ELISA kits in detecting bovine tuberculosis. *Chin J Anim Infect Dis, 24*(05), 29-32. (In Chinese)
26. Zhang, H. J., Wang, H. M., Li, X. H., Zhang, X. L., Liu, G. C., Chen, R. L., & Cao, R. (2016). Analysis of the results of different methods for detecting tuberculosis in dairy cows. *China Anim Health Inspect, 33*(11), 86-88. (In Chinese)
27. Yan, S. G., Wei, Z. J., & Huang, X. W. (2014). Epidemiological Surveillance and Analysis of Dairy Cow Tuberculosis in Liuzhou City. *China Dairy Cattle, 32*(14), 21-23. (In Chinese)
28. Jiang, J. H., Dai, G. W., Wei, B. W., Xie, L. H., Liang, Z., Li, M., & Liu, J. B. (2014). Epidemiological survey of major dairy cattle epidemics in Wuzhou from 2003 to 2013. *J Anim Sci Vet Med, 33*(05), 99-101. (In Chinese)
29. Huang, D. F., Yao, X. J., Liu, J., Wang, T. K., Xue, Y., Wang, R., . . . Ji, C. C. (2016). Detection of tuberculosis in dairy cows in Changping District in 2015. *China Anim Ind, 25*(18), 63. (In Chinese)
30. Wu, X. J., Lu, J., Li, Y., Yang, X. C., Li, K. H., & Zhou, J. P. (2017). Field epidemiological investigation of tuberculosis in a dairy farm in Shanghai. *China Anim Health Inspect, 34*(03), 17-20. (In Chinese)
31. Xu, X. Y., Xu, Z. J., Wang, X. Z., Zhang, C. F., & Chen, C. H. (2014). Analysis of surveillance results of Dairy Cow Tuberculosis in Jiangsu Province from 2007 to 2013. *China Anim Health Inspect, 31*(07), 75-77. (In Chinese)
32. Shen, S. F., Wang, Q. Z., Zhao, H. J., Tang, W. H., & Liu, P. H. (2013). Application of simple intradermal allergy and comparative allergy in detection of bovine tuberculosis. *Chin J Vet Med, 49*(10), 21-24. (In Chinese)
33. Wu, Y. (2015). Detection and analysis of bovine tuberculosis and brucellosis in Jingbian County. *Northwest Agr Forest Univ.* (In Chinese)
34. Lu, G. J., Cao, R., Zhang, J., Liu, N. Q., Tao, M. H., Han, Q. A., . . . Xuan, Q. Y. (2014). Different methods for parallel detection of bovine tuberculosis. *China Anim Health Inspect, 31*(12), 43-46, 74. (In Chinese)
35. Wang, W. Q. (2017). Investigation of tuberculosis infection in dairy farms. *Chin Anim Livest Vet Abstr, 33*(06), 100. (In Chinese)
36. Wang, W. X., Sun, G. L., Li, A. Q., Xu, M., Wang, L. H., Ma, W.P., . . . Li, J. L. (2011). Epidemiological characteristics of tuberculosis between human and livestock in Urumqi. *Grass Feed Livest, 32*(02), 74-76. (In Chinese)
37. Salitanati, J. (2013). Investigation on the infection of bovine tuberculosis to milk around Urumqi. *Xinjiang Agr Univ.* (In Chinese)
38. Sang, C. X., Wang, X. Q., Mulati., Zhang, Q. Y., & Ma, J. (2012). Surveillance and control of bovine tuberculosis in Urumqi county. *Xinjiang Anim Livest, 28*(S1), 22-23. (In Chinese)
39. Shi, Q., Yuan, L. Q., Pu, J. W., Sha, L., & Liu, W. (2018). Epidemiological surveillance of bovine tuberculosis in the suburbs of Urumqi, Xinjiang, 2012-2018. *China Anim Health Inspect, 35*(10), 10-12. (In Chinese)
40. Tan, Q., & Gao, A. X. (2015). Recommendations for the detection and prevention of brucellosis and tuberculosis in dairy cows. *Contemp Anim Livest, 33*(21), 15-16. (In Chinese)
41. Qu, H., Yu, C. R., Gu, X., & Yang, T. (2012). Investigation on tuberculosis of dairy cows in Fengxian area of Shanghai. *China Anim Livest Vet Digest, 28*(04), 97-98. (In Chinese)
42. Ma, D. M. (2017). Investigation of tuberculosis infection in dairy cows. *China Cattle Sci, 43*(03), 87-88. (In Chinese)
43. Zhang, Z. D. (2016). Detection of major infectious diseases in large-scale dairy farms. *Inner Mongolia Agr Univ*. (In Chinese)
44. Zhao, S. J., Zhao, P. D., Liu, X. M., Liu, G. H., Sheng, M., Yuan, S. Y., . . . Ban, F. G. (2019). Survey on tuberculosis infection in large-scale dairy farms in Henan Province. *China Anim Health Inspect, 36*(08), 26-28. (In Chinese)
45. Lin, Y. Q. (2017). Epidemiological investigation of dairy cow brucellosis and tuberculosis in Haidong area of Qinghai. *Gansu Agr Univ.* (In Chinese)
46. Li, S. C. (2010). Epidemiological investigation and diagnosis of bovine tuberculosis. *Xinjiang Agr Univ*. (In Chinese)
47. Liu, C. Y., & Yang, L. C. (2012). Surveillance and survey of dairy cow tuberculosis in Minhe County. *Chin Qinghai J Anim Vet Sci, 42*(02), 55. (In Chinese)
48. Lei, Y. L. (2014). Investigation of tuberculosis infection in dairy cows in Beishan Township, Menyuan County. *J Anim Sci Vet Med, 33*(03), 96, 98. (In Chinese)
49. Li, Z. Y. (2014). Seroepidemiological survey of main infectious diseases of dairy cows in Inner Mongolia. *Inner Mongolia Agr Univ*. (In Chinese)
50. Kong, L. J. (2015). General survey and test of tuberculosis in dairy cows in Liujiaxia Town, Yongjing County. *Vet Orientat, 38*(12), 43, 140. (In Chinese).
51. Gao, Y. Y. (2018). Study on monitoring of tuberculosis in dairy cows of Jingbian Shaanxi. *Northwest Agr Forest Univ.* (In Chinese).
52. Zhou, F. Y. (2013). Tuberculosis and non-tuberculous mycobacteria multiple PCR method to establish and Preliminary application. *Shihezi Univ.* (In Chinese)
53. Hu, Y. J. (2010). Investigation and Research on the Surveillance of "Two Diseases" of Dairy Cows in Minhe County. *China Cattle Sci, 36*(04), 85, 89. (In Chinese)
54. Si, T. D. Y., Xie, Z. Z., & Huang, G. P. (2016). Tuberculosis surveillance report on large-scale dairy farms in Kaiping City. *Chin Livest Poultry Breed, 12*(03), 33-34. (In Chinese)
55. Liu, G. Q. (2019). Incidence and Control Effect of Mastitis and Quarantine Purification of Brucellosis and Tuberculosis in Large-scale Dairy Farm in Reclamation Area. *Northeast Agr Univ.* (In Chinese)
56. Jiang, Y., Wang, Q. Z., Zhang, Y. Y., Shen, S. F., Li, J., Tang, W. H., . . . Shen, X. (2014). Application of rapid detection technology in the detection of tuberculosis in dairy cows. *Chin J Antituberculosis, 36*(6), 447-452. (In Chinese)
57. Wang, L. X., Li, A. H., Wang, H. J., Liu, C. M., Liu, T. J., . . . Han, Q. A. (2015). Comparison of Two Methods for Detection of Bovine Tuberculosis. *China Anim Health, 17*(01), 66-68. (In Chinese)
58. Cheng, W. D. (2010). Investigation on the Epidemic Situation of Dairy Cow's Main Diseases in Changji District, Xinjiang. *Hubei J Anim Vet Sci, 31*(07), 21-22. (In Chinese)
59. Du, P. W. (2017). Report on the detection of brucellosis and tuberculosis in dairy cows in Eryuan County. *Chuna Anim Livest Vet Digest, 33*(09), 125. (In Chinese)
60. Naimaiti, A. (2013). Surveillance and analysis of bovine tuberculosis in Hami City, Xinjiang in the past two years. *Xinjiang Anim Livest, 29*(S2), 26. (In Chinese)
61. Chang, H. S. (2011). General survey and detection of tuberculosis in dairy cows in Tianzhu County. *J Anim Sci Vet Med, 30*(02), 70-71. (In Chinese)
62. Sun, J. W., & Da, J. S. (2016). Comparative study of two methods for detecting tuberculosis in dairy cows. *China Anim Health, 18*(05), 70-73. (In Chinese)
63. Shi, Q., Yuan, L. G., Pu, J. W., Sha, L., & Liu, W. (2018). Comparison of the coincidence rate between two tuberculosis detection methods and PPD detection in dairy cows. *China Anim Health Inspect, 35*(10), 87-89. (In Chinese)
64. Chen, F. M., Cheng, G. M., Ma, A. X., & Hu, S. L. (2016). Serological investigation of BVD-MD, IBR and TB of dairy cows in Weifang and surrounding areas. *Heilongjiang Anim Sci Vet Med, 59*(18), 114-117. (In Chinese)
65. Zhang, S. (2018). Detection and purification of "two diseases" of dairy cows in Beipiao area, Liaoning Province. *China Anim Health, 20*(05), 11-12. (In Chinese)
66. Chen, L. X. (2017). An epidemiological survey of tuberculosis in dairy cows in some areas of Jiyuan City, Henan Province. *China Dairy, 37*(05), 54-55. (In Chinese)
67. Xiong, C. X., & Li, F. X. (2011). Monitoring report of tuberculosis in dairy cows in Midu County. *Yunnan J Anim Sci Vet Med, 40*(02), 21-22. (In Chinese)
68. Song, L. (2019). The epidemiological survey and prevention of tuberculosis in dairy cows in a certain area of Heilongjiang Province. *Feed Rev, 32*(06), 95. (In Chinese)
69. Li, F. S., Zhong, W. D., & Li, Z. H. (2016). Study on the detection of "two diseases" of dairy cows in Minhe County. *Chin Qinghai* *J Anim Vet Sci, 46*(01), 23-24. (In Chinese)
70. Zhang, Q. L., Shen, G. N., Zheng, X. Y., Zhou, D. G., Zhang, W., Liu H. Y., . . . Liu, A. L. (2019). Epidemiological investigation of tuberculosis in a dairy farm in Miyun District, Beijing. *Chin Qinghai J Anim Vet Sci, 36*(07), 20-23. (In Chinese)
71. Ye, L. (2019). Investigation of tuberculosis and brucellosis infection in dairy cows in Guizhou Province and establishment of milk safety evaluation system. *Guizhou Univ.* (In Chinese)
72. La, J. Q. (2010). Investigation of dairy cow tuberculosis in Minhe County. *Qinghai J Anim Livest Vet Medicine*. (In Chinese)
73. Zhang, Q. L. (2010). Report on the general survey of brucellosis and tuberculosis in dairy cows. *Jiangxi J Anim Livest Vet Med*, 29(03), 49. (In Chinese)
74. Xu, F., Tian, L. L., Qi, Y. Y., Sun, S. X., Sun, X. X., Yan, G. Q., . . . Zeng, Q. Y. (2018). Comprehensive diagnosis and pathogen isolation and identification of extrapulmonary tuberculosis in dairy cows. *China Anim Health Inspect*. (In Chinese)
75. He, M. F., Li, H. W., & Ao, Y. P. (2017). Discussion on the application of PPD intradermal allergy detection technology for tuberculosis in dairy cows. *China Anim Health Inspect, 34*(02), 81-83. (In Chinese)
76. Zhang, M., Zhang, J. H. & Han, F. (2012). Establishment and application of PCR detection method for tuberculosis in dairy cows. *Livest Poult Ind, 23*(12), 48-50. (In Chinese)
77. Jin, D. Ch., Wang, Y. Ch., Hu, Y. F., Jin, J. J., & Wang, Z. Y. (2011). Investigation and analysis of epidemic trend of tuberculosis infection in dairy cows. *China Anim Livest Vet Med, 38*(12), 200-202. (In Chinese)
78. Bian, Y., Fang, Ch. L., Lin, H., Tang, H., Zou, L., Dai H., . . . Fu, T. H. (2010). Detection of tuberculosis and brucellosis in dairy cows. *Sichuan Anim Vet Sci, 37*(04), 46. (In Chinese)
79. Song, Z. B., Hou, Y. X., & Guo, J. P. (2019). Clinical study on the detection technology of tuberculosis in dairy cows. *China Dairy Cattle,* *37*(02), 19-21. (In Chinese)
80. Yang, L., Liu, F. Q., Song, D. S., Sun, Q. Y., Tian, M. L., Wu, W. H., . . . Yang, M. S. (2014). Comparison test of two detection methods for tuberculosis in dairy cows. *Guizhou Anim Livest Vet Med, 38*(04), 16-18. (In Chinese)
81. Yang, J. Q., Liang, D. X., & Zhao, M. J. (2013). Comparison of two diagnostic methods for tuberculosis in dairy cows. *Contemp Anim Livest, 31*(33), 22-24. (In Chinese).
82. Wang, Y. M., Ying, Q. X., Zheng, L. Y., & Lu, H. L. (2011). Control of tuberculosis and brucellosis in large-scale dairy farms in Yanping District, Nanping City. *Fujian Anim Livest Vet Med, 33*(01), 11-12. (In Chinese).
83. Zhu, D. D., Sun, Q. Y., Cao, C. G., Chen, J. S., Xu, G, G., Li, K. H., . . . Xue, X. (2011). Comparison of bovine interferon-γ EIA and TST in detecting bovine tuberculosis. *Shanghai J Anim Livest Vet Med, 56*(02), 14-15.
84. Wu, W, H., Yang, M. S., Xu, J. E., & Yang, L. (2014 ). Report on the detection of tuberculosis in a dairy farm with a PCR diagnostic kit for bovine tuberculosis. *Agr Technol Serv, 31*(05), 152, 165. (In Chinese).
85. Zhou, P. X., Li, J. Z., Luan, L. J., Cao, R., Zhang, X. Y., & Li, Y. (2014). The application of ELISA detection method for bovine tuberculosis gamma-interferon in quarantine. *China Anim Health Inspect, 31*(01), 67-71. (In Chinese).
86. Chen, J. (2009). Optimization and application of diagnostic methods for bovine tuberculosis gamma-interferon. *Hunan Agr Univ*. (In Chinese).
87. Wu, W. H., Sun, Q. Y., Yang, M. S., Xu, J. E., & Yang, L. (2014). Detection test of PCR diagnostic kit for bovine tuberculosis. *Shanghai J Anim Livest Vet Med, 59*(02), 56-57, 59. (In Chinese).
88. Wang, X. Q., Li, F. X., Zhao, W. H., Wang, J. P., & Yang, S. B. (2012). Study on detection of two in vivo diagnosis methods for bovine tuberculosis combined with pathogenic diagnosis. *China Animal Health, 14*(04), 10-14, 94. (In Chinese)
89. Yang, X. C., Li, K. H., Wu, X. J., Tao, T. G. S., & Wang, J. (2017). Comparison of skin test method and γ-interferon ELISA method in quarantine of tuberculosis in dairy cows. *Shanghai J Anim Livest Vet Med, 62*(05), 56-57, 59. (In Chinese)
90. Yang, X. C., Wu, X, J., Li, K, H., & Wang, Jian. (2015). Comparison of skin test method and ELISA method in the detection of tuberculosis in dairy cows. *Shanghai J Anim Livest Vet Med, 60*(2), 10-12. (In Chinese)
91. Li, X. M. (2013). Investigation Report on "Two Diseases" in Large-scale Dairy Farms in Qinghai-Tibet Plateau. *Contemp Anim Livest, 31*(30), 23-24. (In Chinese)
92. Lv, W. H. (2011). Investigation on tuberculosis infection of dairy cows in Qiabuqa Town, Qinghai. *Chin J Vet Med,* 47(01):41-42. (In Chinese)
93. Hao, Z. Y., Xing, H. Y., Zhang, J., Wang, L., Kong, L. F., Yin, H. K., . . . Liu, S. D. (2014). Monitoring and purification of tuberculosis and brucellosis in cattle and sheep in Qufu City. *Shandong J Anim Sci Vet Med,* *35*(03), 43-45. (In Chinese)
94. Zhang, X. Y., Lu, G. J., Cao, R., Ni, B., Sun, X. X., Zhang, J., . . . Fan, W. X. (2018). Epidemiological investigation of bovine tuberculosis in a certain area of northern China. *Chin J Prev Vet Med, 40*(04), 350-352.
95. Bing, R. (2013). Serological screening of bovine tuberculosis in China and isolation and identification of its pathogen. *Gansu Agr Univ*. (In Chinese)
96. Chen, Y. Z., Zhang, C. T., Zhen, S. P., Wang, S. Q., & Meng, R. (2014). Investigation on tuberculosis infection of dairy cows in Xining area. *Heilongjiang Anim Sci Vet Med, 57*(10), 72-73. (In Chinese)
97. Zhang, X. H. (2015). Investigation and prevention of tuberculosis in dairy cows in Yinchuan area. *Farmers' wealth consultant, 31*(18), 48-49. (In Chinese)
98. Deng, Y. Q., Yang, A. G., Guo, L., Hou, W., Wen, H., Chen, D., . . . Zhang, D. F. (2014). Comparative study on the application of intradermal allergy and in vitro release test of interferon-gamma to detect tuberculosis in dairy cows. *China Dairy Cattle, 32*(08), 23-25. (In Chinese)
99. Hu, Y. F., Wang, Y. C., Jin, D. C., & Jin, J. J. (2011). Investigation and analysis of tuberculosis infection in dairy cows in Ruian City, Zhejiang Province. *China Dairy Cattle, 32*(07), 44-45. (In Chinese)
100. Ran, X. F. (2018). Epidemiological investigation report of dairy cow brucellosis and tuberculosis in Gongjing District, Zigong City. *Sichuan Agr Univ*. (In Chinese)

**Table D.** Egger’s test for publication bias.

| slope | bias | se. bias | t | df | p-value |
| --- | --- | --- | --- | --- | --- |
| 0.059 | 6.494 | 1.610 | 4.303 | 98 | 0.0001 |

**Table E.** Normal distribution test for the normal rate and the different conversion of the normal rate.

|  | W | P |
| --- | --- | --- |
| PRAW | 0.493 | < 2.2e-16 |
| PLN | NaN | NA |
| PLOGIT | NaN | NA |
| PAS | 0.747 | 7.691e-12 |
| PFT | 0.732 | 3.194e-12 |

PRAW: original rate, PLN, logarithmic conversion, PLOGIT: logit transformation, PAS: arcsine transformation,

PFT: Freeman-Tukey double arcsine transformation, NaN: meaningless number, NA: missing data.
